# Artificial intelligence for dysphagia screening: A machine learning approach

**DOI:** 10.1371/journal.pdig.0001755

**Published:** 2026-09-29

**Authors:** Rafaela Soares Rech, Carla Diniz Lopes Becker, Dieine Estela Bernieri Schiavon, Danielli Pires Vieira, Juliana Balbinot Hilgert, Fernando Neves Hugo

**Affiliations:** 1 Graduate Program in Dentistry, Faculty of Dentistry, Federal University of Rio Grande do Sul, Porto Alegre, Rio Grande do Sul, Brazil; 2 Graduate Program in Information Technology and Healthcare Management, Federal University of Health Sciences of Porto Alegre, Porto Alegre, Rio Grande do Sul, Brazil; 3 Department of Speech-Language Pathology, Federal University of Health Sciences of Porto Alegre, Porto Alegre, Rio Grande do Sul, Brazil; 4 Department of Exact and Applied Social Sciences, Federal University of Health Sciences of Porto Alegre, Porto Alegre, Rio Grande do Sul, Brazil; 5 Department of Preventive and Social Dentistry, Faculty of Dentistry, Federal University of Rio Grande do Sul, Porto Alegre, Rio Grande do Sul, Brazil; 6 Department of Epidemiology and Health Promotion, College of Dentistry, New York University, New York, New York, United States of America; UMC Utrecht: Universitair Medisch Centrum Utrecht, NETHERLANDS, KINGDOM OF THE

## Abstract

Early and reliable screening for oropharyngeal dysphagia symptoms in at-risk populations is a crucial first stage in effective management. This study aims to propose a machine learning model that can predict whether individuals are at risk of having confirmed dysphagia based on diagnostic accuracy. This was an exploratory cross-sectional diagnostic accuracy study. Adults and older adults were screened and assessed, and a videofluoroscopy swallowing study was performed to confirm dysphagia in participants with complaints about swallowing and signs or symptoms identified in the clinical assessment by a speech therapist. Eight algorithms were tested based on a theoretical model to evaluate their diagnostic accuracy: Naive Bayes, K-Nearest Neighbors, Logistic Regression, Decision Tree, Random Forest, Support Vector Machine, Multilayer Perceptron, and Convolutional Neural Networks. The Pandas library in Python was used for data visualization and manipulation. In total, 465 participants were included, of whom 153 had dysphagia. A screening with 15 items related to personal, health, and oral health characteristics is proposed. The final four best-performing models were Naive Bayes (accuracy 0.76, sensitivity 0.84), Logistic Regression (accuracy 0.79, sensitivity 0.82), Support Vector Machine (accuracy 0.78, sensitivity 0.79), and Convolutional Neural Network (accuracy 0.78, sensitivity 0.74). The use of artificial intelligence based on different machine-learning approaches is promising for dysphagia screening, demonstrating adequate diagnostic accuracy. Future studies should be conducted to externally validate the proposed screening models.

## Author summary

Early identification of oropharyngeal dysphagia is critical for clinical management. This study developed and tested machine learning models to predict dysphagia using clinical screening data validated by videofluoroscopy (n = 465). A 15-item screening model achieved accuracies of 0.76–0.79 and sensitivities of 0.74–0.84 across top-performing algorithms. Artificial intelligence shows promise for dysphagia screening, warranting further external validation.

## Introduction

Abnormal swallowing is characterized as dysphagia, a condition that compromises feeding efficiency and safety [1]. Its main consequences include dehydration, malnutrition, aspiration pneumonia, increased hospitalizations, and a higher risk of early mortality [2–5]. Due to its high prevalence in different populations—especially among older adults [6]. and individuals with mechanical lesions, such as cancer [7], or neurological conditions, such as stroke [6] and Parkinson’s disease —and its severe complications, early identification is essential [8]. For this purpose, screening tools have been widely used and validated across various populations and formats, including the Eating Assessment Tool (EAT-10) [9], the Easy Dysphagia Symptom Questionnaire (EDSQ) [10], the Sydney Swallow Questionnaire (SSQ) [11], the MD Anderson Dysphagia Inventory (MDADI) [12], Geriatric Bedside Swallowing Screen (GEBS) [13], Gugging Swallowing Screen (GUSS) [14], and the Ohkuma et al—questionnaire [15].

Despite their availability, the validation of these questionnaires is not always reliable and has been critically analyzed in the literature [16–18]. Moreover, their application and result interpretation often depend on a professional, limiting their usability, especially for older adults, individuals with low educational levels, or visual impairments who may require assistance [19–21]. In this context, new technologies have shown promise in streamlining and improving the dysphagia screening process in hospital, outpatient, and even primary healthcare settings [22]. In recent years, technological advances in using artificial intelligence in health have demonstrated promising potential in detecting and caring for dysphagic patients. Improvements in early diagnosis in a scalable manner help public health managers take more urgent initial measures, always seeking to improve the patient’s clinical condition and, mainly, their well-being in terms of quality of life [10].

Machine learning (ML)-assisted swallowing assessment techniques have shown promising results in detecting post-stroke dysphagia with high sensitivity and specificity [23,24]. Computer-aided screening using ML and deep learning can further enhance dysphagia and aspiration identification in older adults [22]. Recent studies have demonstrated the potential of these methods, applying ML models to bedside screening, videofluoroscopic (VFSS) analysis, and speech-based classification, all with substantial predictive accuracy [22,25,26]. Additionally, algorithms leveraging electronic health records have been developed for dysphagia and aspiration pneumonia detection, marking a step toward clinical implementation [22–24,27]. Despite these advances, further validation is needed using systematic screening data, and more studies are necessary. Future research should refine these models to enable accurate risk prediction and effective dysphagia management in hospitals and other outpatient, clinical, and even primary healthcare settings.

Therefore, the objective of this study is to propose a ML approach that can predict whether individuals are at risk of having confirmed dysphagia based on diagnostic accuracy.

## Materials and methods

### Ethics statement

An exploratory cross-sectional diagnostic accuracy study was conducted in Porto Alegre, Brazil, at the Complexo Hospitalar da Irmandade Santa Casa de Misericórdia. Data collection started in December 2021 and ended in January 2025. The research ethics committee approved the study protocol under number 50257821.9.0000.5345. All participants provided written informed consent prior to participation in the study. The study was conducted in accordance with the principles of the Declaration of Helsinki.

### Reporting guidelines

This study was reported in accordance with the Standards for Reporting Diagnostic Accuracy Studies (STARD 2015) [28]; the completed checklist is provided in S1 File. In addition, the development and reporting of the machine learning models were guided by the TRIPOD+AI recommendations (S2 File) [29].

### Cohort selection

A convenience sample of adults aged 18 years or older was recruited from two sources. The sample included individuals referred for VFSS swallowing assessment and volunteers from the hospital and university communities who reported no swallowing complaints. All participants were invited to undergo the same study procedures.The research team in charge of collecting data underwent theoretical and practical training that covered the established and appropriate format for obtaining informed consent, completing and standardizing the personal identification questionnaire, health characteristics, and behaviors. The theoretical and clinical training lasted 7 hours. Furthermore, a field manual was faithfully followed to collect all variables similarly.

Following consent, the researcher administered a structured questionnaire stored in the Research Electronic Data Capture Software (REDCap) [30] that comprised the following information: age (in years), sex (female, male), BMI(<18.5, 18.5–24.99, and >25), weight loss (yes, no), history of COVID-19 (yes, no), chronic disease (yes, no), chronic obstructive pulmonary disease (yes, no), hypertension (yes, no), osteoporosis (yes, no), diabetes (yes, no), recurrent pneumonia (yes, no), gastroesophageal reflux disease (yes, no), history of cancer (yes, no), history stroke (yes, no) and neurological disease (yes, no), Alzheimer’s (yes, no), Parkinson’s (yes, no), Multiple Sclerosis (yes, no), Amyotrophic Lateral Sclerosis (yes, no), Huntington’s (yes, no), Myasthenia Gravis (yes, no), Machado Joseph (yes, no), Epilepsy (yes, no), Ataxia (yes, no), Sjogren’s Syndrome (yes, no), Dementia (yes, no), Duchenne Syndrome (yes, no), Systemic Sclerosis (yes, no), use of medication with continuous prescription (yes, no), medicines for the digestive system and metabolism (yes, no), medication for the cardiovascular system (yes, no), medicines for the nervous system (yes, no), medication for the respiratory system (yes, no), medicines for the musculoskeletal system (yes, no), smoking (yes, never smoked, former smoker), dry mouth (never, rarely, occasionally, many times, frequently), difficulty swallowing dry food (never, rarely, occasionally, many times, often), teeth in the upper dental arch (fully dentate, partially dentate, edentulous) and teeth in the lower dental arch (fully dentate, partially dentate, edentulous). The variables selected were based on a theoretical model drawn from existing literature [31] Fig 1.

**Fig 1:**
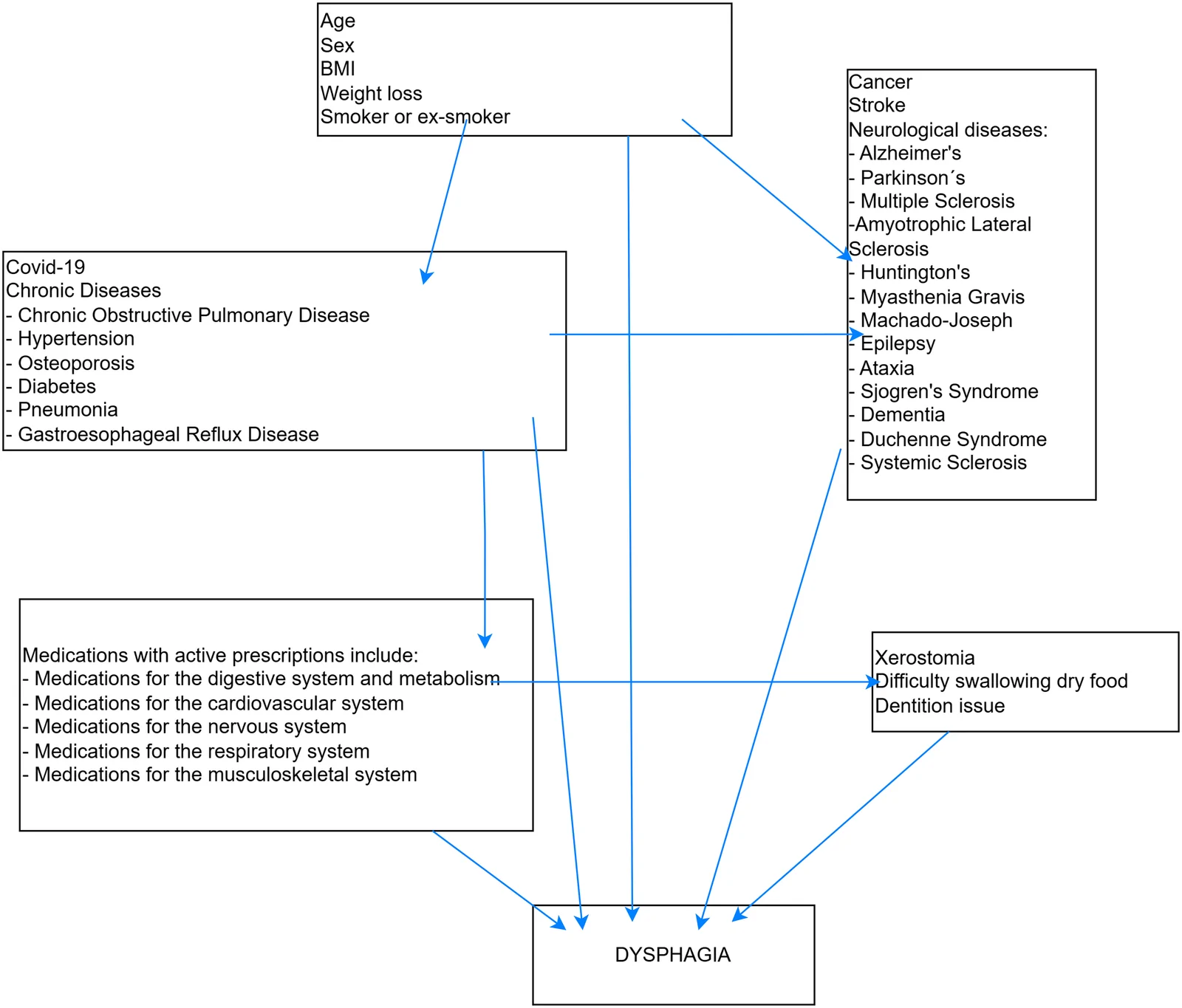
Theoretical model.

To propose a screening, the main variables that could be related to dysphagia were considered and categorized as follows: (18≤age≤55, age > 55), sex (male, female), history of COVID-19 positive (yes, no), chronic disease (yes, no), current or prior cancer (yes, no), stroke (yes, no), neurodegenerative disease (yes, no), use of continuous medication (yes, no), smoking (yes, never smoked, former smoker), dry mouth (never, rarely, occasionally, many times, frequently), dry food (never, rarely, occasionally, many times, often), tooth high arch (fully dentate, partially dentate, edentulous), tooth low arch (fully dentate, partially dentate, edentulous), weight loss (yes, no), BMI (<18.5, 18.5–24.99, and >25).

The primary outcome was the presence of oropharyngeal dysphagia, determined using a prespecified two-stage assessment procedure. Regardless of the recruitment source, all participants underwent the same standardized clinical swallowing assessment. Participants were subsequently classified according to the presence or absence of swallowing complaints, previous clinical or medical suspicion of dysphagia, findings from the standardized clinical assessment, and, when clinically indicated, VFSS findings.

Swallowing was assessed both indirectly and directly. The indirect assessment measured the maximum number of spontaneous saliva swallows performed within one minute. The direct assessment involved the intake of foods and liquids at different consistencies, according to the International Dysphagia Diet Standardisation Initiative (IDDSI) criteria [32]: 200 mL of water (IDDSI level 0), 100 mL of a thickened strawberry-flavored juice preparation (IDDSI level 3), and half a slice of bread (IDDSI level 7). Each consistency was tested under controlled conditions, in which intake was initiated following the speech-language pathologist’s instruction, and under free-intake conditions, in which participants consumed the food or liquid at their own pace.

During the clinical assessment, participants consumed at least three sips of thin liquid, four spoonfuls of the thickened preparation, and three portions of solid food. Oxygen saturation, ambient noise, and swallowing sounds were monitored throughout the assessment. Cervical auscultation was recorded before, during, and after swallowing using an Eko Core amplifier (Eko Health, United States of America) coupled to a 3M Littmann stethoscope (3M, United States of America), positioned lateral to the trachea and immediately below the cricoid cartilage. Following the assessment, the speech-language pathologist completed the Clinical Swallowing Risk Assessment Protocol (PARD) [33]. The protocol assesses variables including anterior oral leakage, oral transit time, nasal reflux, number of swallows, laryngeal elevation, cervical auscultation findings, oxygen desaturation, vocal quality, coughing, choking, and other clinical signs suggestive of swallowing impairment.

Participants recruited from the hospital or university communities who had no self-reported swallowing complaints, no previous clinical suspicion or medical indication for instrumental assessment, and no signs suggestive of dysphagia on the standardized clinical assessment were classified as not having dysphagia for the purposes of this study. Because these participants did not present any clinical indication for instrumental investigation, they were not referred for videofluoroscopic swallowing assessment.

Participants were referred for videofluoroscopy when at least one of the following conditions was present: a prior medical recommendation for instrumental swallowing assessment, a clinical suspicion of dysphagia, signs or symptoms suggestive of swallowing impairment, or findings indicative of dysphagia on the standardized clinical assessment. This included participants who had already been referred for videofluoroscopy as part of their hospital care and participants recruited from the hospital or university communities in whom a possible swallowing impairment was identified during the study assessment. For these participants, the final outcome classification was based on the VFSS findings.

The VFSS swallowing studies were performed at the hospital’s outpatient radiology department by a speech-language pathologist and a radiology technician. The speech-language pathologist responsible for the VFSS assessment was different from the speech-language pathologist who conducted the standardized clinical swallowing assessment.

During the VFSS assessment, participants received thin liquid (IDDSI level 0) through controlled and self-paced cup sips and a homogeneous thickened preparation (IDDSI level 3) administered with a dessert spoon, bread, and crackers. Barium sulfate contrast medium was added to the foods and liquids being tested. The examination was performed with the participant seated and included lateral and anteroposterior views, allowing visualization of bolus transit through the oral, pharyngeal, and esophageal phases of swallowing.

The presence and severity of oropharyngeal dysphagia were classified using the O’Neil Swallowing Scale [34], with dysphagia categorized as mild, mild-to-moderate, moderate, moderate-to-severe, or severe. Videofluoroscopy was therefore used as the reference standard for participants who met the clinical or medical criteria for instrumental swallowing assessment. For participants without swallowing complaints, previous suspicion, medical indication, or clinical signs of swallowing impairment, classification as not having dysphagia was based on the concordant absence of these findings.

The clinical and VFSS assessments were performed independently and under blinded conditions. The speech-language pathologist conducting the standardized clinical assessment was blinded to the VFSS findings. Conversely, the speech-language pathologist and physician responsible for the VFSS interpretation were blinded to the results of the standardized clinical assessment and the ML model outputs. The professionals responsible for the clinical and VFSS assessments did not participate in the development or interpretation of the ML learning models.

The ML models were treated as index tests, and their outputs were generated independently of the clinical and VFSS classifications. When videofluoroscopy was indicated, the index-test data and the reference-standard assessment were obtained within the same clinical assessment period. Participants with incomplete data required for outcome classification or model development were excluded from the analyses.

Access to REDCap data was performed using the system’s API, which allows programmatic extraction of information from projects. The request was made using the POST method with the following main parameters: ‘token’; ‘content’: ‘record’; ‘format’: ‘csv’; ‘type’: ‘flat’ and ‘csvDelimiter.’ The request was sent to the REDCap API using the requests library (in Python). The response was received in the specified format and processed according to the chosen data format (csv). The csv file with the extracted data was saved in a local file. The process ensured efficient and secure access to the REDCap project data, enabling its use for later analysis.

### Preprocessing

The Pandas library in Python was used for data visualization and manipulation. The variables of interest were selected from the DataFrame generated by Pandas, and the data were exported to a new CSV file. The initial dataset comprised 499 participants. Participants without a VFSS report were excluded (n = 22). In the resulting dataset (n = 477), 13 missing values were identified across 12 participants, as one participant had two missing values. After excluding participants with missing data, the final analytical sample consisted of 465 participants. Given the characteristics of the variables, the best approach is to exclude these observations to ensure the data’s quality and integrity for subsequent analyses. The missing data refers to participants without a classification on the O’Neill Scale (n = 8), who were excluded from the analyses, since this scale was used to determine the severity of dysphagia. The absence of O’Neill Scale data was due to the fact that, at the time of compiling the analytical dataset, some videofluoroscopy swallowing studies (VFSS) reports had not yet been issued, making it impossible to classify these participants. The remaining 5 exclusions involved the variables: involuntary weight loss in the last 6 months (n = 1), difficulty with dry foods (n = 2), and dry mouth (n = 2).

After removing missing data, the dataset resulted in 465 instances and 16 columns (15 variables for prediction and the ‘diag’ (target) column with the binary value of the classification 0 for healthy individuals and individuals with functional swallowing, and 1 for dysphagia). Totaling 312 instances with the label 0 and 153 instances with the label 1.

The data was separated into independent variables (X) and dependent (y). The independent variables provide the features or inputs for the ML learning model, while the dependent variable is the output (label) of the model. Next, the first column containing the age values was subjected to the normalization process. For this, StandardScaler was used, a preprocessing technique that transforms the data to ensure a mean equal to zero and a standard deviation equal to one. Due to the wide variation in ages (ranging from 18 to 101 years), age normalization was an important step to ensure that this variable was on the same scale as the other numerical variables, preventing features with greater magnitude from excessively influencing the model during training.

### Machine learning models

The following ML models were evaluated: Naive Bayes (NB), K-Nearest Neighbors (KNN), Logistic Regression (LR), Decision Tree (DT), Random Forest (RF), Support Vector Machine (SVM), Multilayer Perceptron (MLP), and Convolutional Neural Networks (CNN).

NB is a probabilistic algorithm that applies Bayes’ theorem under the “naive” assumption of conditional independence between attributes. The KNN is a simple and intuitive classification algorithm. KNN classifies a new data point based on the majority class of its K nearest training neighbors. The algorithm is sensitive to the value of K, which indicates the number of neighbors considered. The DT constructs a hierarchical tree structure for supervised learning. Each node represents a decision based on an attribute, leading to further splits until a terminal class or value is reached. This transparent structure can handle both categorical and numerical data. LR is a statistical technique used to model the probability of a binary dependent variable. LR is used to predict a probability, which can then be transformed into a binary classification.

RF uses multiple decision trees to improve predictions. Each tree is trained on different random subsets of the data, and their results are combined by voting for classification or averaging for regression, making it an ensemble learning technique. This approach improves accuracy and reduces error. The SVM classifies data by creating hyperplanes to separate classes. MLP consists of one or more hidden layers of neurons and an output layer. The most common architecture is fully connected, where each neuron in a given layer is connected to all neurons in the subsequent layer.

A CNN consists of convolutional and pooling layers that progressively extract and compress relevant features from the input data. As the network deepens, these representations become more complex. The features extracted by CNN are used as input for an MLP, which performs the final classification.

### Training and selection of models

In our experiments, we used eight different machine learning algorithms, from the simplest to the most complex: Naive Bayes (NB), K-Nearest Neighbors (KNN), Logistic Regression (LR), Decision Tree (DT), Random Forest (RF), Support Vector Machine (SVM), Multilayer Perceptron (MLP), and Convolutional Neural Networks (CNN).

The dataset was initially split using the train_test_split method, allocating 25% (n = 117) of the samples for testing and 75% (n = 348) for training. Model performance was then assessed using 5-fold cross-validation (CV) on the training set.

Due to class imbalance, the Synthetic Minority Over-sampling Technique (SMOTE) algorithm [29] was applied to artificially increase the minority class only in the training data. During cross-validation, SMOTE was applied exclusively to the training folds, while the validation folds and the independent test set remained unchanged and contained only real instances to preserve the validity of the model evaluation. After oversampling, the training data became balanced. The independent test set maintained the original class distribution.

During models development, different hyperparameter configurations were evaluated for each algorithm using 5-fold cross-validation on the training set only. For each classifier, a predefined set of candidate hyperparameter values was assessed based on commonly recommended configurations in the literature and exploratory experiments (e.g., k = 1,3,5,7 for the KNN classifier). The combination yielding the highest mean recall across the validation folds was selected, given the study’s screening-oriented nature and the clinical importance of minimizing false negatives. In cases where multiple configurations achieved the same mean recall, the Matthews Correlation Coefficient (MCC) was used as a tie-breaking criterion because it provides a balanced assessment of classification performance in the presence of class imbalance. After hyperparameter optimization, the final model was retrained using the complete training set and subsequently evaluated on the independent test set. A limited predefined search space was adopted for each classifier rather than an exhaustive grid search or randomized search strategy, balancing systematic model optimization with computational efficiency. The final hyperparameter settings for each model are presented in Table 1. The architectural configuration for the CNN is also reported.

**Table 1:** Hyperparameter settings of the final models.

Model	Hyperparameter	Value
NB	var_smoothing	1e-9
	distribution type	‘gaussian’
KNN	n_neighbors	7
	metric	‘euclidean’
	weights	‘distance’
LR	C	1.0
	solver	’lbfgs’
	max_iter	2000
DT	max_depth	3
	min_samples_split	2
	criterion	‘gini’
RF	n_estimators	50
	max_depth	‘None’
	min_samples_split	2
	criterion	‘gini’
SVM	C	5.0
	kernel	‘linear’
	gamma	‘scale’
MLP	hidden_layer_sizes	50
	activation	‘relu’
	solver	‘adam’
	max_iter	1000
CNN	number of convolutional layers	1
	number of filters per layer	16
	filter size	3
	padding size	same
	max pooling filter	2
	hidden layer – output layer	16 (dense) – 1 (output)
	activation function	‘relu’ – ‘sigmoid’
	dropout	0.30 (conv) - 0.40 (dense)
	L2 regularization	0.001
	loss function	binary_crossentropy
	optimizer	‘adam’ – lr = 0.001
	batch size	32
	max epochs	200
	early stopping	val_loss – patience = 12
	epochs executed	64

NB, Naive Bayes; KNN, K-Nearest Neighbors; LR, Logistic Regression; DT, Decision Tree; RF, Random Forest; SVM, Support Vector Machine; MLP, Multilayer Perceptron; CNN, Convolutional Neural Networks.

In health, decisions based on predictive models can directly impact people’s lives, so the choice of evaluation metrics is of fundamental importance. Two metrics widely used in this context are accuracy and sensitivity (recall). The metrics used to evaluate the performance of the models were recall, accuracy, and their respective means and standard deviations obtained during cross-validation. Sensitivity measures a model’s ability to correctly identify the true positive instances. It is the ratio of true positives to the sum of true positives and false negatives. Accuracy evaluates the proportion of correct predictions. The metrics were used to assess the models’ performance, and the final metric for evaluating the best models was based on obtaining the best results in sensitivity, specificity, F1 Score, and MCC obtained in the test data. Specificity assesses the method’s ability to correctly identify negative results, i.e., the proportion of true negatives among all real negatives. The F1 score is a ML evaluation metric that harmoniously combines precision and recall, providing a balance between the two. The MCC is a statistical metric that evaluates the prediction quality considering all categories of the confusion matrix [35]. Its value ranges from -1 to +1, where +1 indicates a perfect prediction, 0 is a random prediction, and -1 is a poor prediction.

### Sensitivity analysis

To assess the potential impact of verification bias, a sensitivity analysis was conducted by restricting the evaluation to participants with VFSS-confirmed outcomes (n = 290). Model performance was evaluated both with and without SMOTE using the same hyperparameters selected in the primary analysis (see S3 File).

### Interpretability

We use interpretability techniques to understand the contributions of features to the decision process, ensuring transparency and confidence in model predictions. We apply the LIME (Local Interpretable Model-agnostic Explanations) [36] method to the model CNN. The technique provides local explanations of the model’s predictions. Global interpretability was assessed using Permutation Feature Importance [37] for the LR, SVM, NB and CNN models, identifying influential variables by measuring decreased model sensitivity after permuting features. This combined approach offers both detailed and broad insights into model performance.

## Results

In total, 465 participants were included, of whom 153 (32.9%) were confirmed to have dysphagia by videofluoroscopy. The participant flow diagram based on STARD recommendations is presented in Fig 2, detailing participant enrollment, clinical assessment, VFSS referral, exclusions, missing data, and final diagnostic classification. Most of the sample (n = 284, 62.4%) was over 55 years old, and 275 (59.1%) were female. Tables 2–4 shows the sample characteristics stratified by the presence or absence of oropharyngeal dysphagia. Among those diagnosed, the most common degree of dysphagia was mild, 57 (37.3%). The majority, 129 (84.3%), had a history of chronic diseases, 49 (32.0%) had been diagnosed with cancer, and 30 (19.6%) with stroke. The majority of the sample (n = 135, 88.2%) reported continuous medication use. In addition, 60 (39.2%) individuals reported difficulty eating dry foods frequently, and the majority were partially dentate in the maxilla (n = 77, 50.3%) and the mandible (86, 56.2%). In the sample without dysphagia, the majority (n = 159, 51.0%) had functional swallowing at the time of assessment. In addition, 184 (59.0%) individuals had a diagnosis of some chronic disease, and 203 (65.0%) reported continuous medication use. Dry mouth sensation was considered rare by 187 (60.0%), and the majority of the sample was fully dentate in the maxilla (n = 187, 59.9%) and the mandible (n = 187, 59.9%).

**Fig 2:**
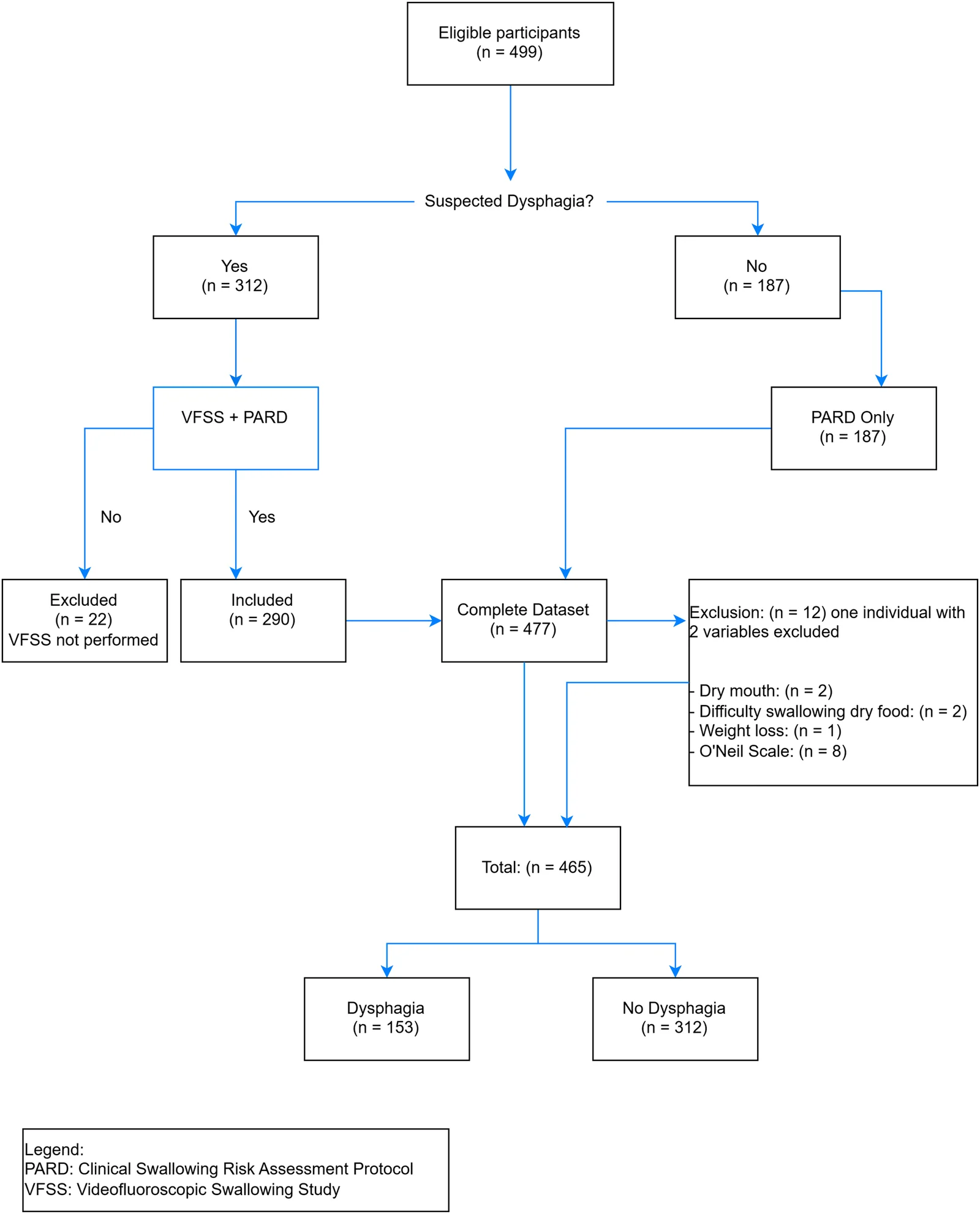
Participant flow diagram based on STARD recommendations.

**Table 2:** Socio-demographic and clinical characteristics stratified by oropharyngeal dysphagia status.

Variable	Total, n (%)	Normal/Functional, n (%)	Dysphagia, n (%)
**Age (years)**			
≥18 age ≤55	218 (46.9)	188 (60.2)	30 (19.6)
age > 55	247 (53.1)	124 (39.8)	123 (80.4)
**Sex**			
Male	190 (40.9)	107 (34.3)	83 (54.2)
Female	275 (59.1)	205 (65.7)	70 (45.8)
**History of Covid-19**			
Positive (Yes)	189 (40.6)	142 (45.5)	47 (30.7)
Negative (No)	276 (59.4)	170 (54.5)	106 (69.3)
**Chronic disease**			
Yes	313 (67.3)	184 (59.0)	129 (84.3)
No	152 (32.7)	128 (41.0)	24 (15.7)
**Current/Prior Cancer**			
Yes	92 (19.8)	43 (13.8)	49 (32.0)
No	373 (80.2)	269 (86.2)	104 (68.0)
**Stroke**			
Yes	46 (9.9)	16 (5.1)	30 (19.6)
No	419 (90.1)	296 (94.9)	123 (80.4)
**Neurodegenerative**			
Yes	67 (14.4)	27 (8.7)	40 (26.1)
No	398 (85.6)	285 (91.3)	113 (73.9)
**Continuous Medication**			
Yes	338 (72.7)	203 (65.0)	135 (88.2)
No	127 (27.3)	109 (35.0)	18 (11.8)
**Smoking**			
Yes	27 (5.8)	19 (6.1)	8 (5.2)
Never Smoked	308 (66.2)	227 (72.8)	81 (53.0)
Former Smoker	130 (28.0)	66 (21.1)	64 (41.8)

**Table 3:** Oral health, feeding difficulties, and nutritional status stratified by dysphagia status.

Variable	Total, n (%)	Normal/Functional, n (%)	Dysphagia, n (%)
**Dry mouth**			
Never/Rarely	250 (53.8)	187 (60.0)	63 (41.2)
Occasionally	84 (18.0)	54 (17.3)	30 (19.6)
Many times/Frequently	131 (28.2)	71 (22.7)	60 (39.2)
**Dry food difficulty**			
Never/Rarely	258 (55.5)	215 (69.0)	43 (28.1)
Occasionally	55 (11.8)	35 (11.1)	20 (13.1)
Many times/Frequently	152 (32.7)	62 (19.9)	90 (58.8)
**Teeth (upper dental arch)**			
Fully dentate	222 (47.7)	187 (59.9)	35 (22.9)
Partially dentate	180 (38.7)	103 (33.0)	77 (50.3)
Edentulous	63 (13.6)	22 (7.1)	41 (26.8)
**Teeth (lower dental arch)**			
Fully dentate	224 (48.2)	187 (59.9)	37 (24.2)
Partially dentate	198 (42.6)	112 (35.9)	86 (56.2)
Edentulous	43 (9.2)	13 (4.2)	30 (19.6)
**Weight loss**			
Yes	151 (32.5)	75 (24.0)	76 (49.7)
No	314 (67.5)	237 (76.0)	77 (50.3)
**BMI category**			
< 18.5	30 (6.5)	10 (3.3)	20 (13.1)
18.5 - 24.99	182 (39.1)	118 (37.8)	64 (41.8)
> 25	253 (54.4)	184 (58.9)	69 (45.1)

BMI = Body Mass Index.

**Table 4:** Detailed distribution of the sample according to the O’Neill Dysphagia Severity Scale.

O’Neill Scale Level	Total, n (%)	Normal/Functional, n (%)	Dysphagia, n (%)
Level I: Severe Dysphagia	10 (2.1)	–	10 (6.5)
Level II: Moderate/Severe	16 (3.4)	–	16 (10.5)
Level III: Moderate	40 (8.6)	–	40 (26.1)
Level IV: Mild/Moderate	30 (6.5)	–	30 (19.6)
Level V: Mild Dysphagia	57 (12.3)	–	57 (37.3)
Level VI: Functional	159 (34.2)	159 (51.0)	–
Level VII: Normal	153 (32.9)	153 (49.0)	–
**Total**	**465 (100)**	**312 (100)**	**153 (100)**

Analyses of the initial tests are described in Table 5. When analyzing the eight proposed models to predict the presence of dysphagia, it was observed that the majority had adequate results for screening. MLP worst, with a sensitivity of 0.58 and an accuracy of 0.72. The four outstanding models were NB (sensitivity 0.84, accuracy 0.76), LR (sensitivity 0.82, accuracy 0.79), SVM (sensitivity 0.79, accuracy 0.78), and CNN (sensitivity 0.74, accuracy 0.78). Although the CNN model achieved the lowest sensitivity among these models, it presented the highest specificity (0.80) and the lowest false-positive rate (0.20). Table 6 presents the confusion matrices of the four best-performing models. The NB model correctly identified 32 positive cases (84%) and 57 negative cases (72%) but also produced 22 false positives (28%) and six false negatives (16%). The LR model correctly identified 31 positive cases (82%) and 61 negative cases (77%) but resulted in 18 false positives (23%) and seven false negatives (18%). The SVM model correctly identified 30 positive cases (79%) and 61 negative cases (77%), producing 18 false positives (23%) and eight false negatives (21%). The CNN model correctly identified 28 positive cases (74%) and 63 negative cases (80%) but resulted in 16 false positives (20%) and ten false negatives (26%).

**Table 5:** Performance evaluation of initial models. Brazil, 2021-2025.

Model	Sensitivity Mean CV	Sensitivity Std CV	Accuracy Mean CV	Accuracy Std CV	Test Sensitivity	Test Accuracy
NB	0.74	0.05	0.74	0.06	0.84	0.76
KNN	0.75	0.06	0.70	0.04	0.71	0.73
LR	0.78	0.03	0.77	0.04	0.82	0.79
DT	0.63	0.05	0.69	0.03	0.63	0.72
RF	0.78	0.03	0.64	0.11	0.63	0.74
SVM	0.80	0.04	0.79	0.07	0.79	0.78
MLP	0.74	0.04	0.65	0.06	0.58	0.72
CNN	0.82	0.08	0.80	0.04	0.74	0.78

Naive Bayes (NB), K-Nearest Neighbors (KNN), Logistic Regression (LR), Decision Tree (DT), Random Forest (RF), Support Vector Machine (SVM), Multilayer Perceptron (MLP), Convolutional Neural Networks (CNN), Cross Validation (CV), Standard Deviation (Std).

**Table 6:** Confusion matrices of the best-performing models on the test set, 2021–2025.

Model	True Positive (TP)	True Negative (TN)	False Positive (FP)	False Negative (FN)
NB	32 (0.84)	57 (0.72)	22 (0.28)	6 (0.16)
LR	31 (0.82)	61 (0.77)	18 (0.23)	7 (0.18)
SVM	30 (0.79)	61 (0.77)	18 (0.23)	8 (0.21)
CNN	28 (0.74)	63 (0.80)	16 (0.20)	10 (0.26)

Logistic Regression (LR), Support Vector Machine (SVM), Naive Bayes (NB), Convolutional Neural Networks (CNN).

Table 6 presents the confusion matrix of the four best-performing models. The NB model correctly identified 32 positive cases (84%) and 57 negative cases (72%) but also produced 22 false positives (28%) and six false negatives (16%). The LR model achieved 31 (82%) true positives and correctly classified 61 negative cases (77%), 18 false positives (23%), and seven false negatives (18%). The SVM model correctly identified 30 positive cases (79%), 61 negative cases (77%), 18 false positives (23%), and eight false negatives (21%). The CNN model correctly identified 28 (74%) true positive cases and 63 (80%) true negative cases, achieved the lowest number of false positive cases, 16 (20%), and the highest number of false negatives, 10 (26%).

The final comparison of test-set performance metrics is presented in Table 7. NB achieved the highest recall (0.84), with a specificity of 0.72, accuracy of 0.76, precision of 0.59, F1 score of 0.70, and MCC of 0.53. LR demonstrated the most balanced overall performance, with a sensitivity of 0.79, specificity of 0.77, accuracy of 0.79, precision of 0.63, F1 score of 0.71, and MCC of 0.56. It achieved the highest accuracy, F1 score, and MCC among the evaluated models. The SVM yielded a sensitivity of 0.79, specificity of 0.77, accuracy of 0.78, precision of 0.63, F1 score of 0.70, and MCC of 0.53. The CNN achieved the highest specificity (0.80) and precision (0.64), with an accuracy of 0.78, but presented the lowest sensitivity (0.74), F1 score (0.68), and MCC (0.52).

**Table 7:** Test set performance metrics for final model selection. Brazil, 2021–2025.

Model	Recall	Specificity	Accuracy	Precision	F1 Score	MCC
NB	0.84	0.72	0.76	0.59	0.70	0.53
LR	0.82	0.77	0.79	0.63	0.71	0.56
SVM	0.79	0.77	0.78	0.63	0.70	0.53
CNN	0.74	0.80	0.78	0.64	0.68	0.52

Naive Bayes (NB), Logistic Regression (LR), Support Vector Machine (SVM), Convolutional Neural Networks (CNN), Matthews Correlation Coefficient (MCC).

These findings demonstrate a trade-off between the ability to detect dysphagia and overall classification balance. LR provided the best overall performance according to accuracy, F1 score, and MCC. In contrast, NB achieved the highest sensitivity and the lowest false-negative rate. Given the intended application as a screening instrument, in which missed positive cases are considered more clinically consequential than false-positive classifications, NB represents the more conservative screening approach. Nevertheless, its lower specificity may increase the number of individuals referred for confirmatory assessment. LR may therefore be preferred when a more balanced compromise between sensitivity and specificity is required.

The results of the sensitivity analysis are summarized in Table 8. Overall model performance in the VFSS-confirmed cohort was lower than that observed in the primary analysis, consistent with the substantially smaller sample size (290 vs. 477 participants). Differences between the analyses with and without SMOTE were generally modest. Logistic Regression and Decision Tree achieved the highest MCC values in the VFSS + SMOTE scenario, whereas Logistic Regression showed the highest MCC without SMOTE. Although CNN achieved the highest sensitivity without SMOTE, it was accompanied by a substantial reduction in specificity.

**Table 8:** Sensitivity analysis restricted to the VFSS-confirmed cohort (*n* = 290).

Model	VFSS + SMOTE					VFSS without SMOTE				
	Sens	Spec	Acc	MCC	AUC	Sens	Spec	Acc	MCC	AUC
LR	0.63	0.69	0.66	0.46	0.75	0.66	0.70	0.68	0.35	0.71
SVM	0.55	0.73	0.63	0.28	0.70	0.55	0.70	0.62	0.25	0.69
NB	0.61	0.61	0.61	0.21	0.65	0.63	0.61	0.62	0.24	0.66
CNN	0.63	0.67	0.65	0.30	0.70	0.87	0.33	0.62	0.24	0.70
RF	0.66	0.58	0.62	0.23	0.71	0.66	0.58	0.62	0.23	0.70
DT	0.79	0.67	0.73	0.46	0.75	0.63	0.70	0.66	0.33	0.73
KNN	0.63	0.67	0.65	0.30	0.67	0.68	0.58	0.63	0.26	0.66
MLP	0.55	0.45	0.51	0.01	0.63	0.53	0.55	0.54	0.07	0.65

Sensitivity (Sens), Specificity (Spec), Accuracy (Acc), Matthews Correlation Coefficient (MCC), Area Under the Receiver Operating Characteristic Curve (AUC), Logistic Regression (LR), Support Vector Machine (SVM), Naive Bayes (NB), Convolutional Neural Networks (CNN), Random Forest (RF), Decision Tree (DT), K-Nearest Neighbors (KNN), Multilayer Perceptron (MLP), Synthetic Minority Oversampling Technique (SMOTE), Videofluoroscopic Swallowing Study (VFSS).

LIME was applied to all models with a true-positive case Fig 3. Because Convolutional Neural Networks are less directly interpretable than traditional machine-learning models, LIME was applied to representative CNN predictions, including true-positive, false-positive, and false-negative cases, to investigate local decision patterns and potential failure modes. In Figs 4–6, it is possible to observe that dysphagia prediction is shown for true-positive, false-positive, and false-negative cases.

**Fig 3:**
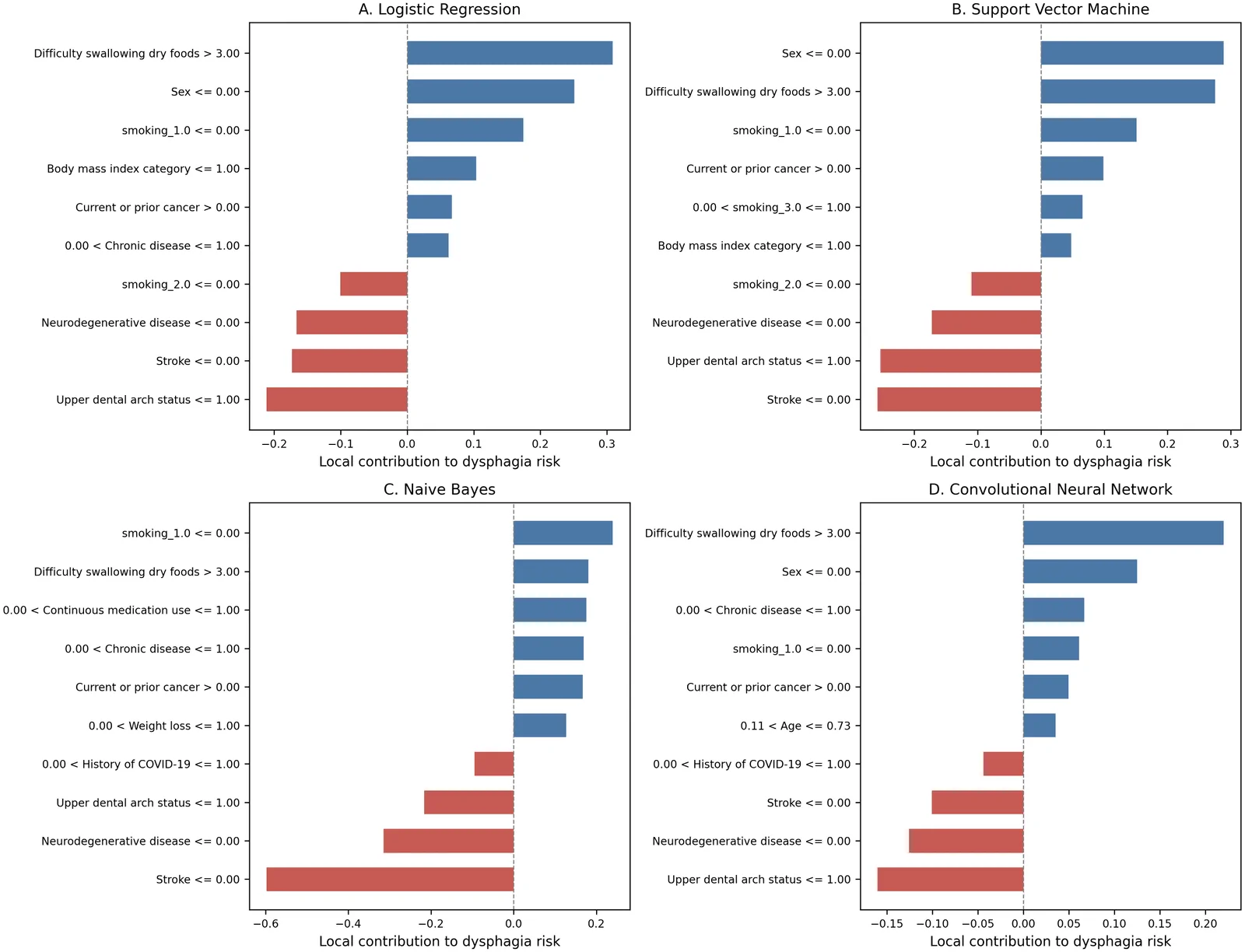
LIME explanations for representative true-positive predictions of the four best-performing models for dysphagia screening. Red bars indicate features contributing toward dysphagia prediction, whereas blue bars indicate contributions toward no dysphagia.

**Fig 4:**
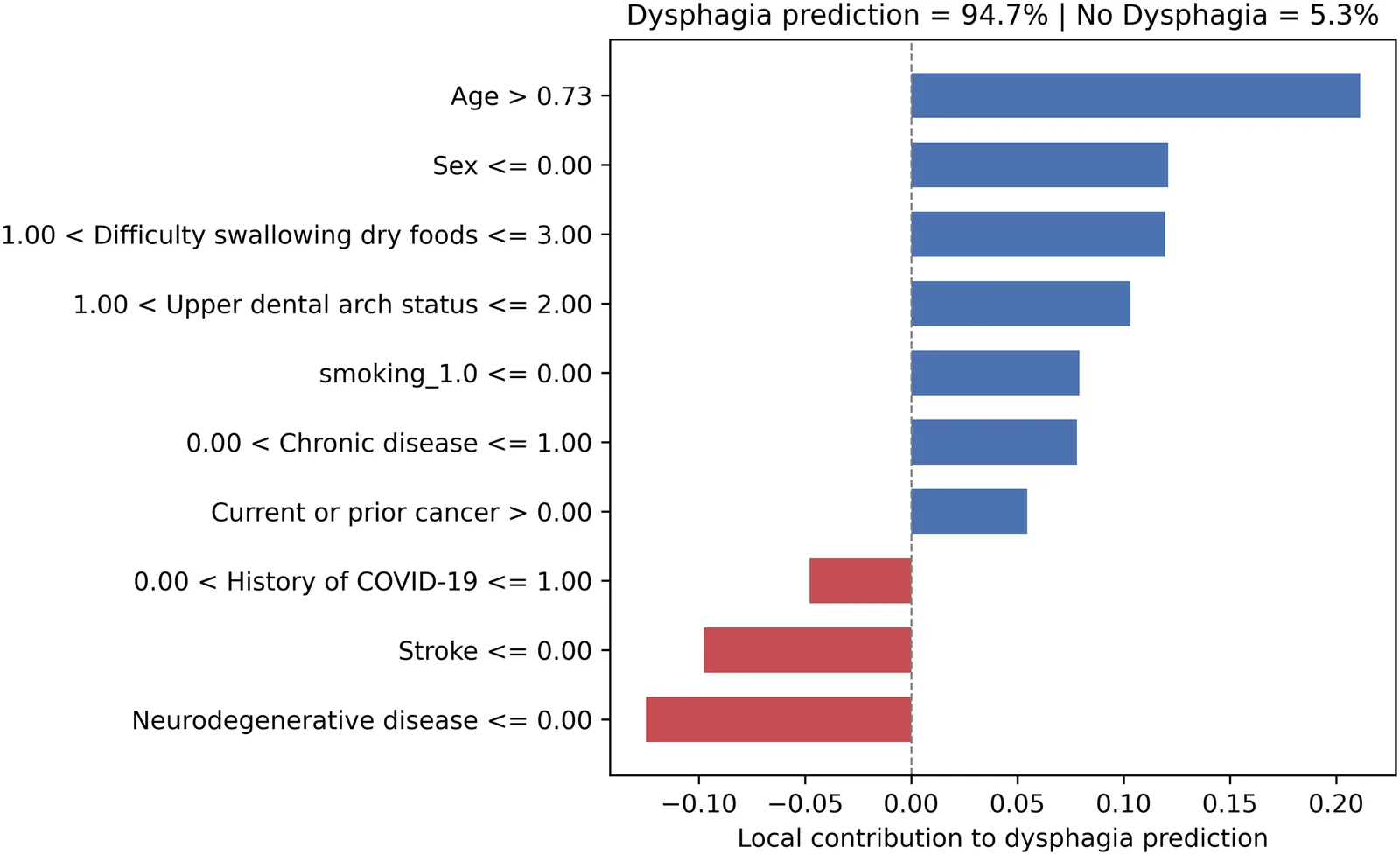
LIME explanation for a true-positive CNN prediction for dysphagia.

**Fig 5:**
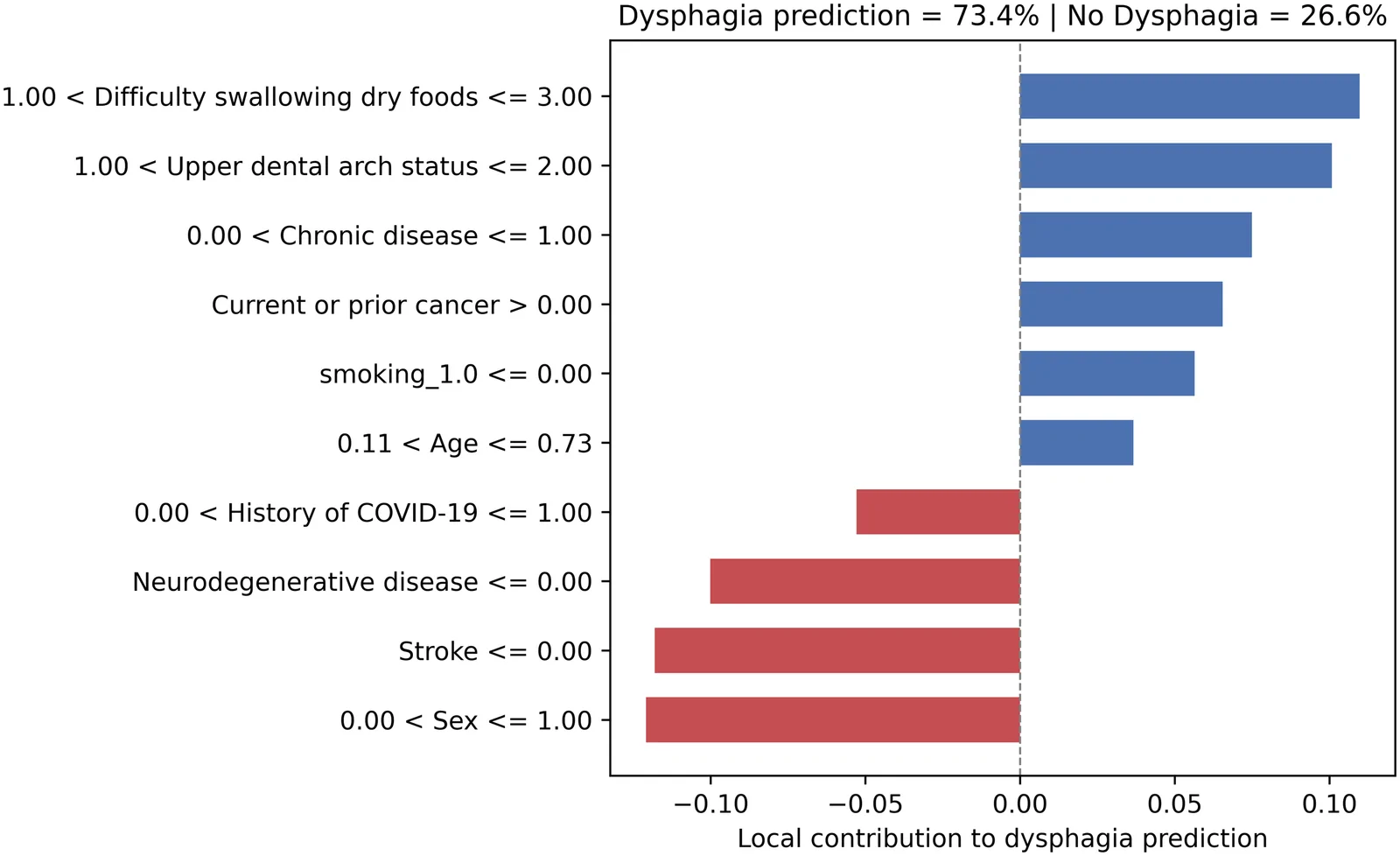
LIME explanation for a false-positive CNN prediction for dysphagia.

**Fig 6:**
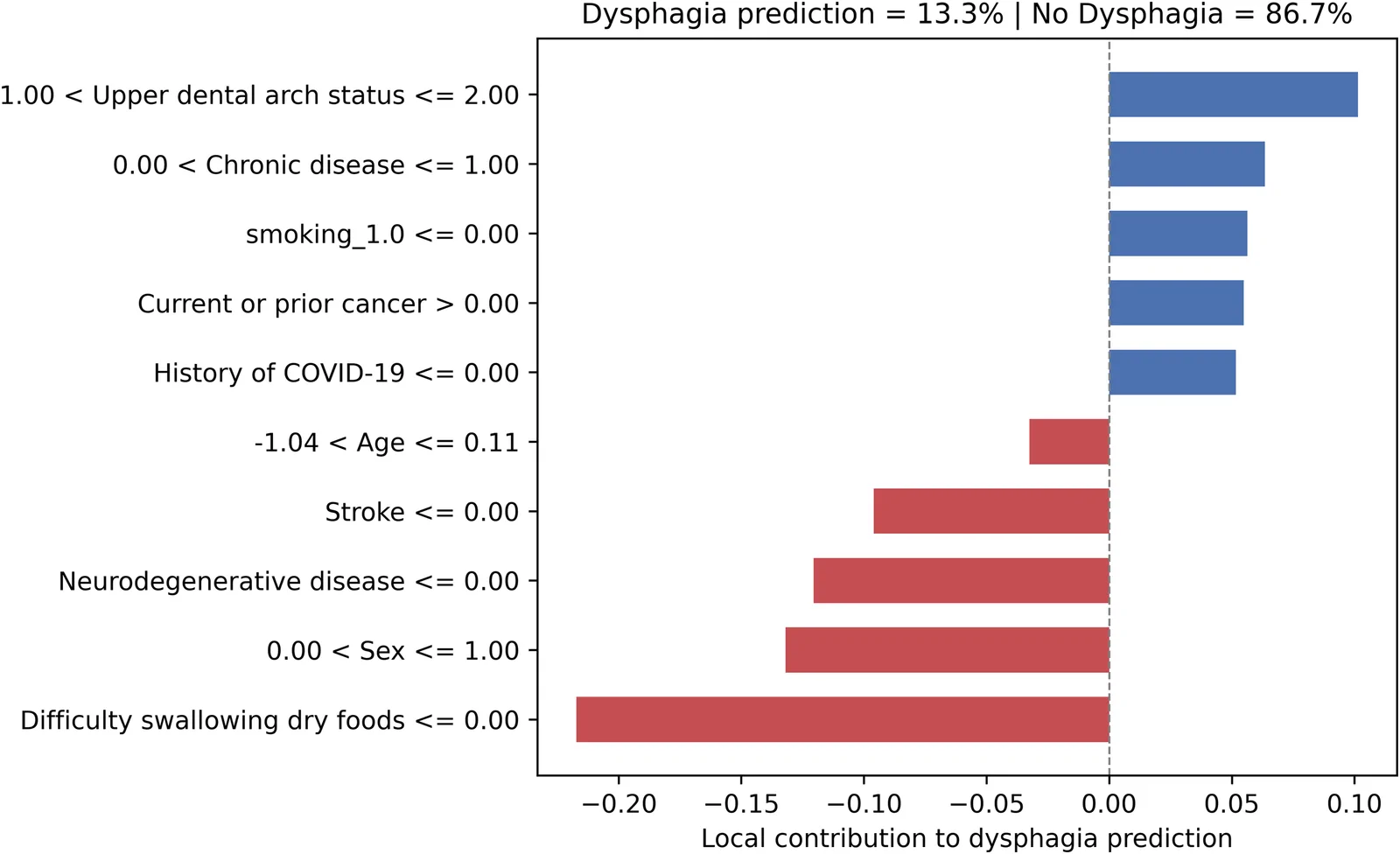
LIME explanation for a false-negative CNN prediction for dysphagia.

Permutation Feature Importance(PFI) analysis was performed separately for each best-performing model using 100 random permutations per feature. Feature importance was quantified as the mean decrease in either the MCC or sensitivity after feature permutation, and the results are shown in Figs 7 and 8.

**Fig 7:**
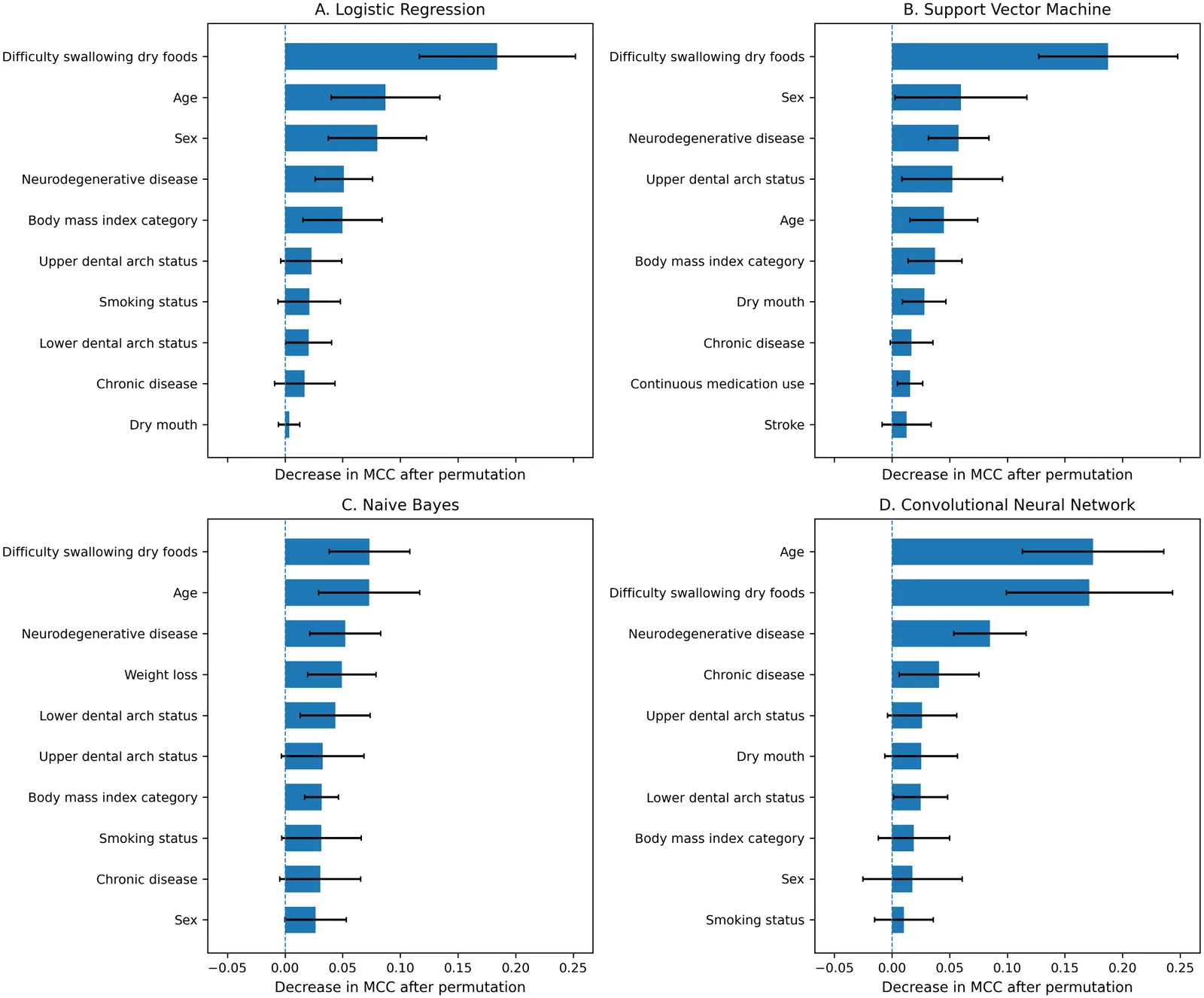
Permutation Feature Importance based on the decrease in Matthews Correlation Coefficient (MCC). Feature importance was estimated separately for the best-performing Logistic Regression **(A)**, Support Vector Machine **(B)**, Naive Bayes **(C)**, and Convolutional Neural Network (D) models. Bars represent the mean decrease in MCC after feature permutation, and error bars indicate the standard deviation across 100 permutations.

**Fig 8:**
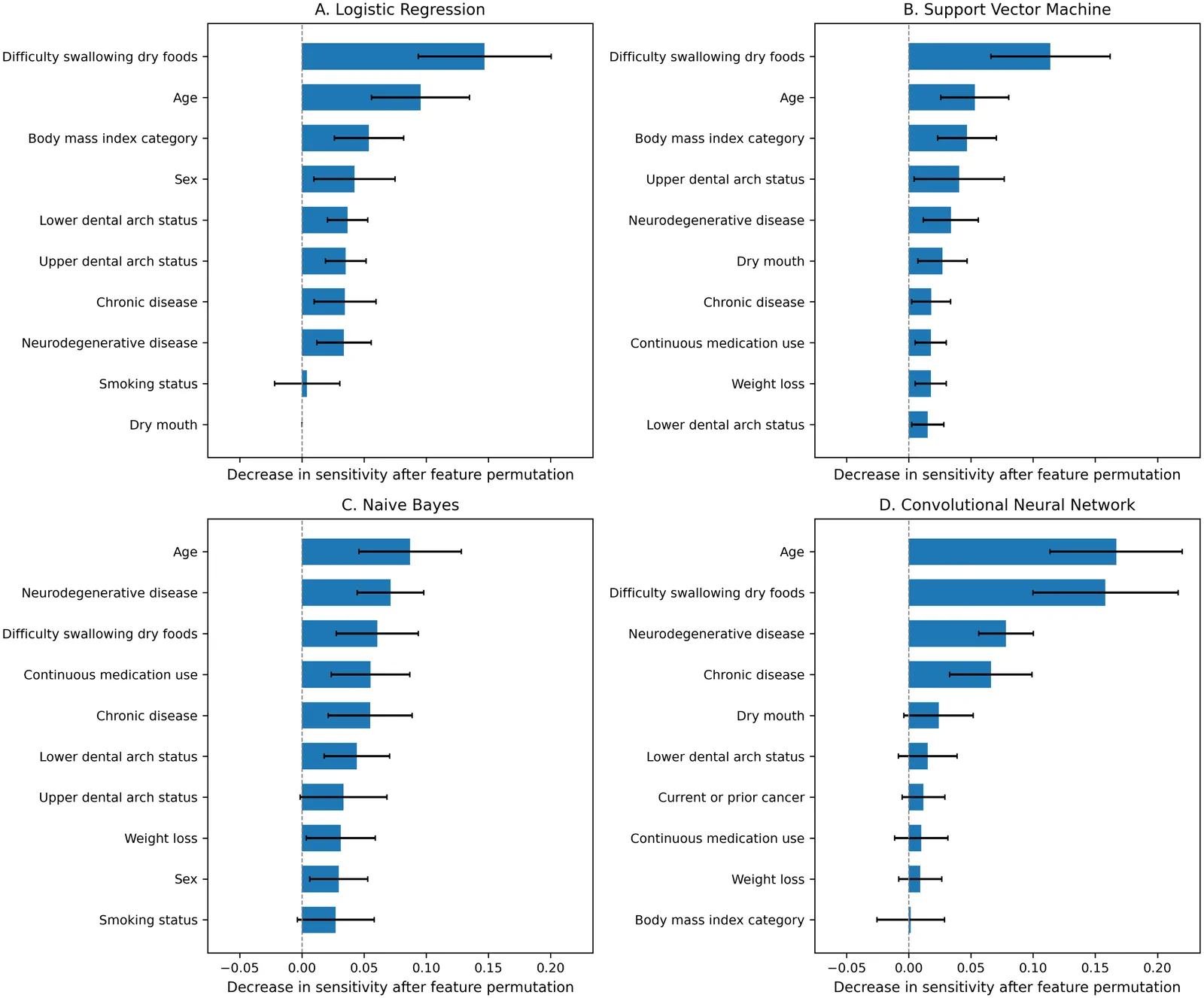
Permutation Feature Importance based on the decrease in sensitivity. Feature importance was estimated separately for the best-performing Logistic Regression **(A)**, Support Vector Machine **(B)**, Naive Bayes **(C)**, and Convolutional Neural Network (D) models. Bars represent the mean decrease in sensitivity after feature permutation, and error bars indicate the standard deviation across 100 permutations.

PFI based on sensitivity yielded results largely consistent with those obtained using MCC. Across all classifiers, difficulty swallowing dry foods, age, and neurodegenerative disease remained among the most influential predictors, indicating robust feature relevance regardless of the evaluation metric. Minor differences were observed for Naive Bayes, in which age became the most influential predictor when sensitivity was used, suggesting that this classifier relies more heavily on age to correctly identify positive cases. Overall, the stability of the rankings supports the robustness of the identified predictors and strengthens the interpretability of the proposed models.

## Discussion

The use of artificial intelligence, particularly ML, has shown promise in screening adults and older adults for dysphagia. This study identified 15 key items related to personal characteristics (age, sex, weight, height, and weight loss), health conditions (diseases and medication use), and oral health (number of remaining teeth and xerostomia). These items can be easily self-reported and applied by professionals from various fields, not limited to healthcare, and in different settings. The LR model demonstrated superior overall performance among the evaluated models, achieving the highest accuracy (0.79), F1 score (0.71), and MCC (0.56), with a sensitivity of 0.82 and specificity of 0.77. In contrast, the CNN yielded more modest results, with a sensitivity of 0.74, accuracy of 0.78, F1 score of 0.68, and MCC of 0.52, although it achieved the highest specificity (0.80). Although the NB model did not achieve the best overall performance, it showed the highest sensitivity (0.84), which is particularly important in healthcare screening applications where minimizing false-negative cases is a priority. These findings reinforce the potential of the four evaluated models as promising screening tools, although further validation studies are still required, for the early identification of individuals who may require further swallowing evaluation.

Although the LR model demonstrated the best overall performance across multiple evaluation metrics, including accuracy, F1 score, and MCC, it exhibited slightly lower sensitivity than the NB model. In the context of dysphagia screening, particularly from a public health perspective, sensitivity is a critical parameter, as false negatives may lead to serious clinical consequences, such as aspiration pneumonia, malnutrition, and increased morbidity. In this sense, simpler models such as NB, which achieved the highest sensitivity, may represent a more appropriate choice when prioritizing the early identification of individuals at risk. However, the CNN achieved the highest specificity, indicating a lower proportion of false-positive classifications. Therefore, model selection should be guided by the intended application, balancing overall performance with clinical priorities and the potential impact of diagnostic errors.

When comparing our study with previous research, it is notable that different predictive models for dysphagia have been developed, often focused on specific populations or clinical settings. For example, Jauk et al. (2023) [22] proposed a ML-based tool using clinical variables routinely collected in hospital environments, such as neurological diseases, history of pneumonia, nutritional status, functional dependence, and cognitive ability [22]. Similarly, Tomita et al. (2018) developed a predictive scale with traditional statistics based on objective VFSS swallowing study findings, focusing on markers such as penetration-aspiration, pharyngeal residue, and laryngeal reflux to estimate the risk of aspiration pneumonia in individuals with Parkinson’s disease [27]. Both approaches significantly contribute to the field by improving risk stratification and supporting clinical decisions. Yet, they require access to specialized evaluations—whether through comprehensive clinical assessments or instrumental examinations—which may limit their routine use, particularly in resource-constrained settings.

In addition to these models with traditional statistics, specific tools have also been created for post-stroke populations, such as the PreDyScore, designed to assess the risk of dysphagia and assist in decisions regarding percutaneous endoscopic gastrostomy placement during the short- and medium-term recovery phases [22], and the Royal Adelaide Prognostic Index for Dysphagic Stroke (RAPIDS), which aims to predict prolonged dysphagia in acute stroke patients by integrating clinical and neurological variables like stroke severity, level of consciousness, presence of aspiration, and nutritional markers [38]. While clinically valuable, these tools depend on detailed in-hospital evaluations and are restricted to a particular diagnostic context. In contrast, our study proposes a model based on simple, self-reported variables related to personal characteristics, health conditions, and oral health, which can be easily obtained without needing specialized resources. This design has the potential to broaden the applicability of the screening tool, allowing its use by professionals from diverse fields and in varied settings, including community and primary care, thereby enhancing opportunities for early identification of individuals at risk for dysphagia and facilitating timely intervention across multiple levels of healthcare. Additionally, Park et al. (2023) [23] developed a ML-based predictive model to screen for aspiration risk in hospitalized patients with acute stroke using a dataset of clinical variables such as age, sex, stroke subtype, neurological status, and laboratory findings [23]. Their model, trained and validated within an acute care environment, showed high accuracy in identifying patients at risk for aspiration, highlighting the potential of artificial intelligence to support complex clinical decision-making during hospitalization. However, despite its promising performance, the model relies on detailed clinical and laboratory data and the immediate availability of healthcare infrastructure typical of hospital settings. Such requirements may limit its generalizability to outpatient, community, or primary care contexts.

In contrast, our approach emphasizes using easily obtainable self-reported variables, aiming to provide a complementary strategy that broadens access to dysphagia risk screening beyond specialized environments. By prioritizing simplicity and feasibility, our model seeks to address gaps in early detection, particularly in resource-limited settings where comprehensive clinical evaluations are not always accessible. From an epidemiological perspective, an effective screening tool should prioritize high sensitivity to minimize the likelihood of false negatives, ensuring that individuals at risk are accurately identified and referred for further evaluation or intervention [39]. In the context of dysphagia, missing cases can result in serious complications, such as aspiration pneumonia and malnutrition, reinforcing the need for tools that err on the side of inclusion rather than exclusion. While specificity is essential to avoid unnecessary referrals, sensitivity is often considered the most critical attribute for initial screening instruments, mainly when applied in large or diverse populations [28]. Accordingly, our model was designed to emphasize maximizing sensitivity, aligning with established public health principles for screening strategies.

Integrating artificial intelligence into screening processes offers new possibilities for improving the early detection of health conditions, particularly in large populations and settings with limited access to specialized care. In screening strategies, where high sensitivity is essential to minimize missed cases, AI can process complex combinations of variables and identify subtle patterns that may not be easily captured through traditional methods [40].

ML models based exclusively on self-reported information may help identify adults more likely to require a comprehensive swallowing assessment. Because these models use variables that can be collected without specialized equipment, they may represent an accessible approach to preliminary risk stratification in outpatient and community settings. However, their results should not be interpreted as a diagnosis of dysphagia or as a substitute for a standardized clinical swallowing assessment or instrumental examination when clinically indicated. Moreover, the effects of using these models on referral pathways, access to care, demand for specialized services, and patient outcomes were not evaluated in the present study and should be investigated before their implementation in clinical practice.

This study has limitations. First, no formal a priori sample size calculation was performed due to the study’s exploratory nature. The sample size was determined by the number of eligible participants recruited during the study period. Although the number of participants classified as having dysphagia enabled an exploratory assessment of model performance, future validation studies should determine the required sample size based on the expected prevalence of dysphagia, sensitivity and specificity, the number of candidate predictors, and the desired precision of the estimates. Second, convenience sampling may have introduced selection bias and limited the generalizability of the findings. Participants were recruited from two sources: adults receiving outpatient care who had been referred for VFSS swallowing assessment and volunteers from the hospital and university communities. The study did not include individuals receiving emergency care or patients with severe or medically unstable conditions. Therefore, the sample primarily represents adults assessed in outpatient and community settings and may not reflect the full clinical spectrum of dysphagia. In particular, the performance of the models in individuals with acute illness, severe dysphagia, complex medical conditions, or a high risk of aspiration remains unknown. The absence of these populations may also have reduced the variability and severity of dysphagia represented in the sample.

Another important limitation concerns outcome ascertainment. VFSS was not performed on all participants. Individuals with a previous medical recommendation for instrumental swallowing assessment, a clinical suspicion of dysphagia, signs or symptoms suggestive of swallowing impairment, or abnormal findings on the standardized clinical swallowing assessment were referred for VFSS. Conversely, participants without self-reported swallowing complaints, previous clinical suspicion or medical indication, and signs of dysphagia on the standardized clinical assessment were classified as not having dysphagia without undergoing VFSS. Thus, dysphagia status was determined through a two-stage procedure, and the same reference-standard assessment was not applied to the entire sample. To reduce the risk of outcome misclassification among participants who did not undergo VFSS, the assessment considered their known health conditions and clinical history, including previous pneumonia, unexplained weight loss, medical conditions associated with swallowing impairment, and other potential indicators of dysphagia or aspiration. This information was analyzed together with the absence of self-reported swallowing complaints and the absence of abnormal findings on the standardized clinical swallowing assessment. The concordant absence of complaints, relevant clinical indicators, and signs of swallowing impairment reduced the likelihood of clinically unrecognized dysphagia in these participants. Nevertheless, silent aspiration cannot be definitively excluded without instrumental assessment. Therefore, some participants with mild, subclinical, or silent swallowing impairment may have been incorrectly classified as not having dysphagia. Although the clinical assessment and review of relevant health indicators were intended to minimize this possibility, they do not eliminate the potential for partial or differential verification bias. As a methodological strength, sensitivity analyses restricted to participants who underwent VFSS were performed to examine the robustness of the findings under complete reference-standard verification. The overall consistency of the diagnostic performance estimates between the primary and sensitivity analyses supports the stability of the findings across the two analytic approaches. However, these analyses quantify the potential impact of verification bias rather than eliminate it, because referral for VFSS remained clinically indication-driven. The reported model performance estimates should therefore be interpreted with appropriate caution.

The study was conducted at a single hospital in southern Brazil, which further limits the applicability of the findings to populations with different clinical, sociodemographic, healthcare, and epidemiological characteristics. Data collection occurred between December 2021 and January 2025, a period partially influenced by the COVID-19 pandemic. Although the proportion of participants reporting COVID-19 infection was low and few required hospitalization, the possibility that the pandemic influenced recruitment patterns or the characteristics of the study population cannot be entirely excluded.

Despite these limitations, the study has relevant strengths. All participants underwent the same standardized clinical swallowing assessment based on a validated protocol. When videofluoroscopy was clinically indicated, the clinical and instrumental assessments were conducted independently by different speech-language pathologists. The speech-language pathologist responsible for the clinical assessment was blinded to the VFSS findings. Conversely, the speech-language pathologist and physician responsible for the VFSS assessment and interpretation were blinded to the clinical classification and ML outputs. The professionals responsible for the clinical and VFSS assessments did not participate in the development or interpretation of the ML models.

Another strength was the incorporation of interpretability analyses into the ML models. In addition to evaluating predictive performance, local explanation plots and permutation feature importance analyses were used to examine the contribution of individual variables to model predictions. Difficulty swallowing dry foods, neurodegenerative disease, age, history of stroke, history of cancer, and oral health conditions consistently contributed to predictions across different models. Local explanation plots also demonstrated how combinations of variables increased or decreased the predicted probability of dysphagia in correctly classified, false-positive, and false-negative cases. These analyses improved model transparency and enabled examination of the clinical plausibility of the predictors. Nevertheless, interpretability does not establish clinical validity or eliminate the need for external validation [29,36].

The contribution of this study lies in the exploratory development of ML models based exclusively on self-reported variables to identify adults who may require further swallowing assessment. The proposed approach does not introduce a novel ML technique and should not yet be considered ready for clinical implementation. Rather, it provides preliminary evidence regarding the feasibility of using readily obtainable information to support risk stratification and indicate the need for subsequent assessment by a speech-language pathologist.

## Conclusion

ML models based on self-reported variables showed potential to identify adults in outpatient and community settings who may warrant further clinical swallowing assessment. However, the findings should be considered exploratory because of the convenience sample, the exclusion of hospitalized and emergency-care patients, the single-center design, and the two-stage outcome ascertainment procedure in which videofluoroscopy was not performed in all participants. Although a standardized clinical assessment and a review of relevant health conditions and clinical indicators were used to reduce the risk of outcome misclassification, silent aspiration could not be completely excluded among participants who did not undergo instrumental assessment. These models should not replace clinical or instrumental swallowing assessments. Prospective, multicenter studies involving larger, more heterogeneous samples, including participants across varying levels of dysphagia severity and healthcare settings, are required to externally validate the models before their clinical or population-level implementation.

## Supporting information

S1 File Supplementary material 1 - STARD. (PDF)

S2 File Supplementary material 2 - TRIPOD+AI. (PDF)

S3 File Sensitivity analysis for the VFSS-confirmed cohort (n = 290). (PDF)
