# Supplementary material for: Artificial intelligence for dysphagia screening: A machine learning approach
Source: PLOS Digit Health. 2026 Sep 29;5(9):e0001755. doi: 10.1371/journal.pdig.0001755 (PMC13623117; doi:10.1371/journal.pdig.0001755)
Supplement: Supplementary file 3 [file pdig.0001755.s003.pdf]

S3. Sensitivity Analysis for the VFSS-Confirmed Cohort (n = 290)

This supplementary material presents the performance metrics of all evaluated models, receiver operating characteristic (ROC) and precision–recall (PR) curves for the sensitivity analysis restricted to participants with VFSS-confirmed outcomes (n = 290). Results are reported both with and without the application of the Synthetic Minority Oversampling Technique (SMOTE).

1. With SMOTE

Model	Accuracy	Sensitivity	Specificity	Precision	F1-score	MCC	ROC AUC	PR AUC (AP)
Decision Tree	0.7324	0.7895	0.6667	0.7317	0.7595	0.4606	0.7520	0.7248
Logistic Regression	0.6620	0.6316	0.6970	0.7059	0.6667	0.3280	0.7065	0.7700
KNN	0.6479	0.6316	0.6667	0.6857	0.6575	0.2975	0.6711	0.6851
CNN	0.6479	0.6316	0.6667	0.6857	0.6575	0.2975	0.6962	0.6715
SVM	0.6338	0.5526	0.7273	0.7000	0.6176	0.2826	0.6954	0.7572
Random Forest	0.6197	0.6579	0.5758	0.6410	0.6494	0.2342	0.7109	0.7786
Naive Bayes	0.6056	0.6053	0.6061	0.6389	0.6216	0.2108	0.6451	0.7179
MLP	0.5070	0.5526	0.4545	0.5385	0.5455	0.0072	0.6340	0.7353

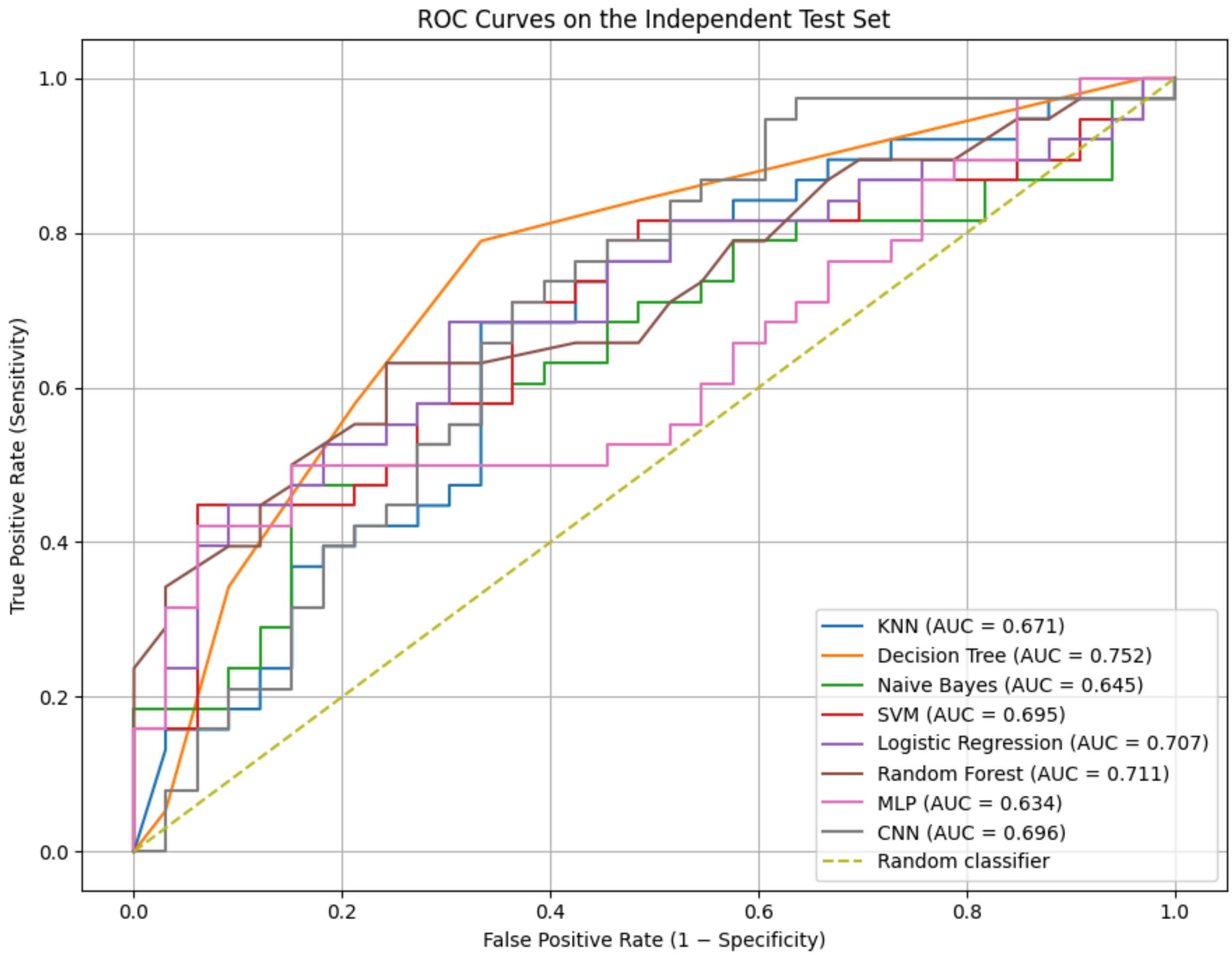

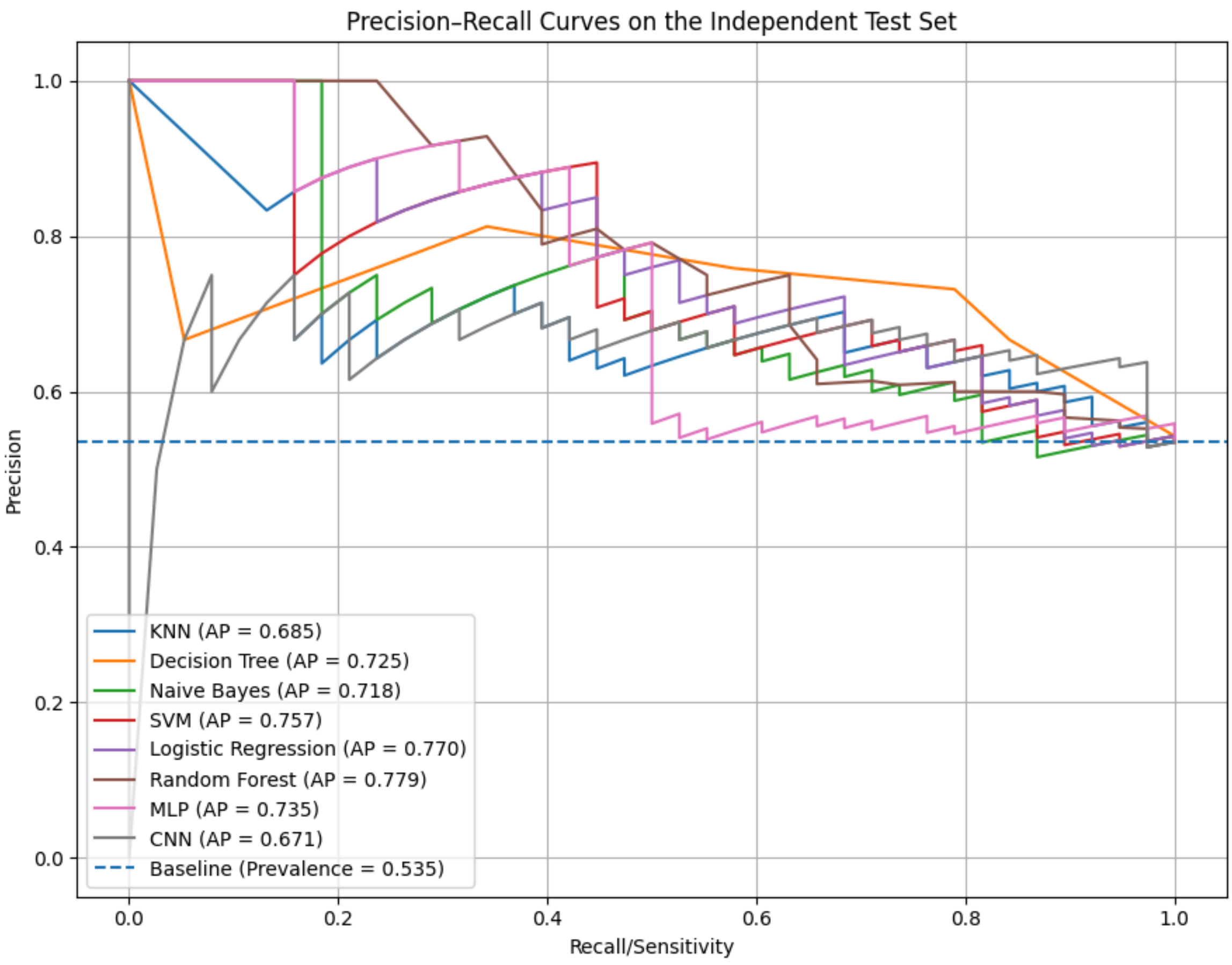

2. Without SMOTE

Model	Accuracy	Sensitivity	Specificity	Precision	F1-score	MCC	ROC AUC	PR AUC (AP)
**Logistic Regression**	0.6761	0.6579	0.6970	0.7143	0.6849	0.3540	0.7081	0.7797
**Decision Tree**	0.6620	0.6316	0.6970	0.7059	0.6667	0.3280	0.7281	0.7220
**KNN**	0.6338	0.6842	0.5758	0.6500	0.6667	0.2614	0.6575	0.6827
**SVM**	0.6197	0.5526	0.6970	0.6774	0.6087	0.2510	0.6946	0.7574
**CNN**	0.6197	0.8684	0.3333	0.6000	0.7097	0.2408	0.7010	0.7723
**Naive Bayes**	0.6197	0.6316	0.6061	0.6486	0.6400	0.2373	0.6555	0.7336
**Random Forest**	0.6197	0.6579	0.5758	0.6410	0.6494	0.2342	0.6962	0.7510
**MLP**	0.5352	0.5263	0.5455	0.5714	0.5479	0.0716	0.6507	0.7459

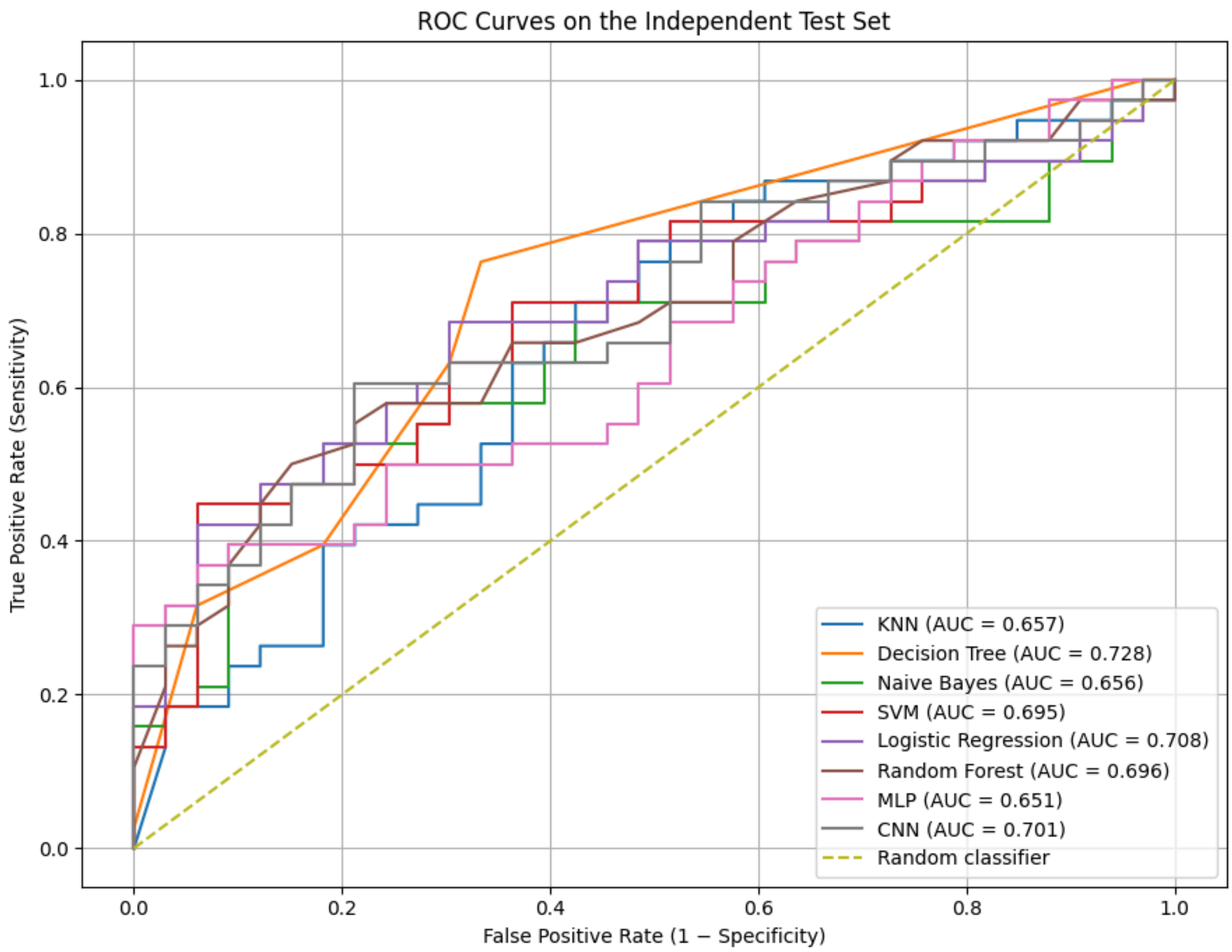

ROC Curves on the Independent Test Set
True Positive Rate (Sensitivity)
False Positive Rate (1 − Specificity)
0.0
0.2
0.4
0.6
0.8
1.0
KNN (AUC = 0.657)
Decision Tree (AUC = 0.728)
Naive Bayes (AUC = 0.656)
SVM (AUC = 0.695)
Logistic Regression (AUC = 0.708)
Random Forest (AUC = 0.696)
MLP (AUC = 0.651)
CNN (AUC = 0.701)
Random classifier

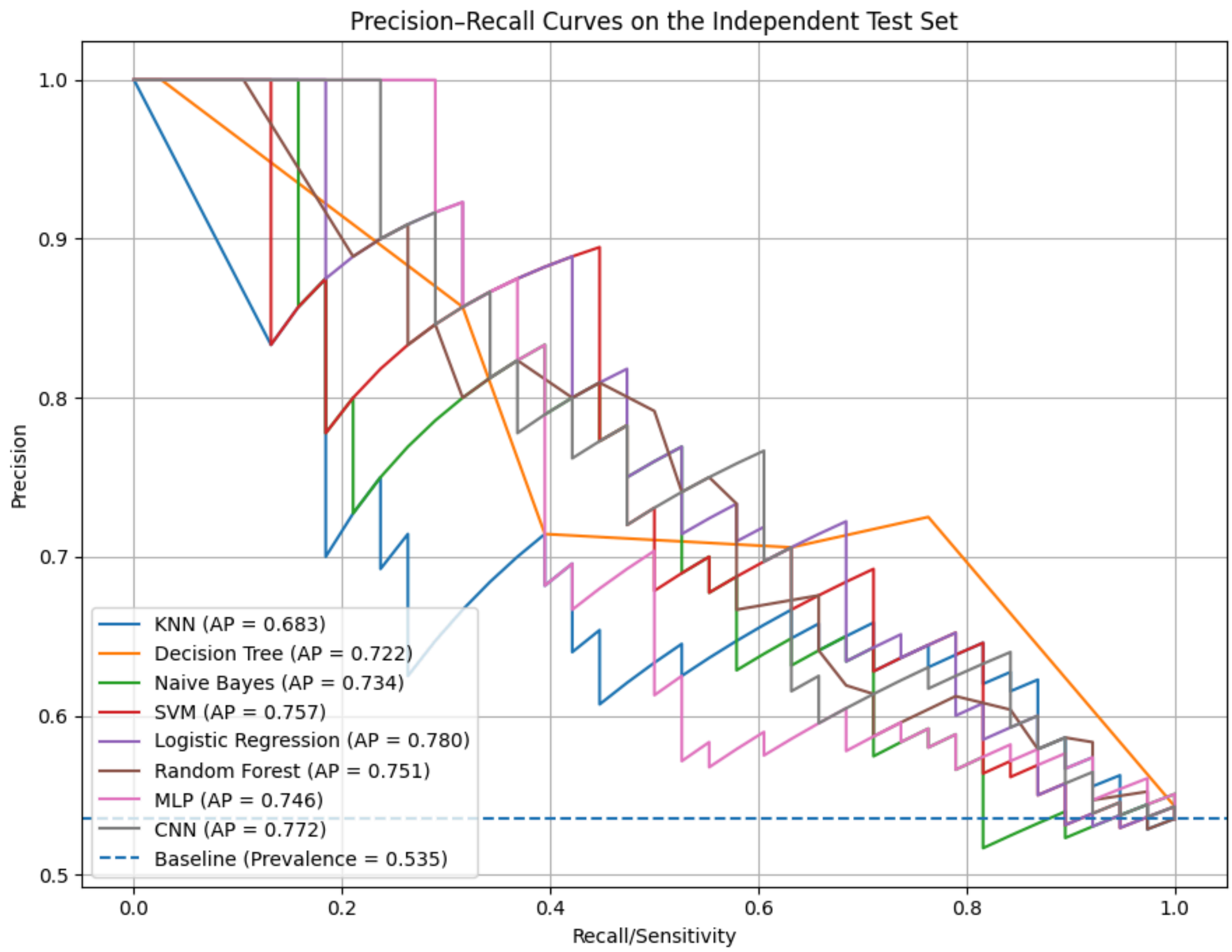

Precision–Recall Curves on the Independent Test Set
Precision
Recall/Sensitivity
0.5
0.6
0.7
0.8
0.9
1.0
0.0
0.2
0.4
0.6
0.8
KNN (AP = 0.683)
Decision Tree (AP = 0.722)
Naive Bayes (AP = 0.734)
SVM (AP = 0.757)
Logistic Regression (AP = 0.780)
Random Forest (AP = 0.751)
MLP (AP = 0.746)
CNN (AP = 0.772)
Baseline (Prevalence = 0.535)
