# Supplementary material for: Artificial intelligence for dysphagia screening: A machine learning approach
Source: PLOS Digit Health. 2026 Sep 29;5(9):e0001755. doi: 10.1371/journal.pdig.0001755 (PMC13623117; doi:10.1371/journal.pdig.0001755)
Supplement: Supplementary file 4 [file pdig.0001755.s005.pdf]

Manuscript PDIG-D-26-00067

Dear,

Leo Anthony Celi
Editor-in-Chief
PLOS Digital Health

We sincerely thank the reviewers for their comments and suggestions regarding the proposed changes to the scientific article. We value all comments received, as they contribute to the quality of our publication and strengthen the focus on artificial intelligence and dysphagia screening methods.

We have incorporated the reviewers' suggestions into the previously submitted manuscript and performed a comprehensive review of the material to increase clarity and coherence. The most relevant changes are highlighted in red throughout the text.

We are available for further clarification.
All authors.

Response to Additional Editor Comments

Editor Comment: "Please specifically address the critical issue raised by Reviewer 1 on the erroneous citation."

Response: We have addressed this point with priority. The erroneous citation mentioned by Reviewer 1 was corrected to *Gandolfo C, Sukkar S; on behalf of the PreDyScore Group; et al. The predictive dysphagia score (PreDyScore) in the short- and medium-term post-stroke: a putative tool in PEG indication. Neurol Sci. 2019;40(8):1619–1626*. We also re-verified all other references in that section to ensure accuracy.

Response to Reviewers

Reviewer #1: General Comments

This manuscript presents a diagnostic accuracy study evaluating machine learning algorithms for dysphagia screening using 15 self-reported variables. While the topic is clinically relevant and the sample size (n=455) is adequate for exploratory analysis, several critical methodological and ethical issues require substantial revision before consideration for publication.

Answer:

1. **Critical Issue: Fictitious or AI-Generated Citation**

Reference 32 contains severe inaccuracies that suggest either fabrication or excessive reliance on AI-generated content without verification.

Manuscript citation:

Malandraki GA, Markaki V, Georgopoulos VC, Kapsalakis G, et al. The predictive dysphagia score (PreDyScore) in the short- and medium-term post-stroke: a putative tool in PEG indication. Eur J Phys Rehabil Med. 2016;52(2):207–216.

Verified correct citation:

Gandolfo C, Sukkar S; on the behalf of the PreDyScore Group; Ceravolo MG, Cortinovis F, Finocchi C, Gradaschi R, Orlandoni P, Reale N, Ricci S, Vassallo D, Zini A. The predictive dysphagia score (PreDyScore) in the short- and medium-term post-stroke: a putative tool in PEG indication. Neurol Sci. 2019 Aug;40(8):1619-1626. doi: 10.1007/s10072-019-03896-2. PMID: 31004231.

Required actions:

Authors must provide documentation of how this erroneous citation was generated

Complete audit of all references to verify accuracy

Explanation in the response letter regarding this discrepancy

Answer: *We thank the reviewer for identifying this critical issue. We fully acknowledge the importance of ensuring the accuracy and traceability of all references.*

The error in Reference 23 resulted from manual handling of citations during the early stages of manuscript preparation, prior to the use of a reference management system. This process led to inconsistencies in author attribution and bibliographic details. We emphasize that this was an unintentional error and not related to the use of AI-generated references or any form of fabrication.

To address this issue, we conducted a comprehensive audit of the entire reference list, which included:

- *Importing all references into Zotero*
- *Verifying metadata using DOI and PMID identifiers*
- *Manually cross-checking each reference against its original publication*

Specifically, Reference 23 has been corrected to the verified citation provided by the reviewer:
Gandolfo C, Sukkar S; on behalf of the PreDyScore Group; et al. The predictive dysphagia score (PreDyScore) in the short- and medium-term post-stroke: a putative tool in PEG indication. Neurol Sci. 2019;40(8):1619–1626.

We confirm that all references in the revised manuscript have undergone this verification process, ensuring their accuracy and reliability.

Additionally, to improve transparency, we carefully reviewed all in-text citations to ensure that each statement is appropriately supported by the cited evidence.

We appreciate the reviewer's attention to this issue, which has significantly strengthened the methodological rigor of our manuscript.

We also provide below an example of how we performed the citation review based on the references used in the material:

1. *McCarty EB, Chao TN. Dysphagia and Swallowing Disorders. Med Clin North Am. setembro de 2021;105(5):939–54. doi:10.1016/j.mcna.2021.05.013*
    a. *Paragraph quoted in the introduction: Abnormal swallowing is characterized as dysphagia, a condition that compromises feeding efficiency and safety (1).*
    b. *Paragraph used as the basis for the quotation: "Dysphagia is a symptom of swallowing impairment that can occur if there is a malfunction with any part of the swallowing mechanism. Dysphagia can reduce quality of life, can compromise nutrition, and may result in penetration or aspiration of oropharyngeal secretions or food contents into the airway, compromising ventilation".*

2. **Overstated Innovation Claim**

The manuscript claims to be "innovative in its approach"; however, similar studies using machine learning for dysphagia prediction have been published (e.g., Jauk et al., 2023; Park et al., 2023). The contribution should be reframed as a community-based screening tool using self-reported data rather than a novel methodological approach. This distinction is important for accurate positioning within the existing literature.

Answer: *We thank the reviewer for this thoughtful comment. We agree that machine learning approaches for dysphagia prediction have been previously reported, including studies based on clinical and hospital-derived datasets. However, we respectfully emphasize that the innovation of our study does not lie in developing a novel machine learning algorithm, but rather in the conceptual and practical design of the screening approach.*

Specifically, our study introduces:

- *A screening model based exclusively on self-reported variables that can be obtained through a simple anamnesis, without requiring clinical examination, instrumental assessment, or access to hospital infrastructure*
- *A low-complexity and scalable framework designed for use in community and primary care settings*
- *A shift from clinically dependent and resource-intensive models toward a feasible, population-level screening strategy*

While prior studies (e.g., Jauk et al., 2023; Park et al., 2023) rely on structured clinical data, neurological assessments, laboratory findings, or instrumental measures, our approach is intentionally designed to operate in non-specialized contexts, where such resources are often unavailable. Therefore, we believe that the innovation of this study lies in translating machine learning into a simplified, accessible screening tool, rather than in methodological novelty per se.

To address the reviewer's concern, we have revised the manuscript to clarify this distinction by:

- *Refining the wording to avoid implying novelty in the machine learning methodology itself*
- *Explicitly emphasizing the pragmatic and context-driven innovation of the approach*
- *Better positioning the study within the existing literature*

We believe this clarification strengthens the scientific positioning of our work while preserving its original contribution.

Add in the discussion:
"reinforcing its potential as a promising screening tool, although further validation studies are required, and early identification of individuals who may require further swallowing evaluation."

"In any case, this study is innovative in its approach, translating machine learning into a simplified screening model based exclusively on self-reported variables, enabling the identification of individuals at risk of dysphagia through a scalable, accessible approach applicable to non-specialized settings. Rather than introducing a novel machine learning technique, this approach emphasizes feasibility and applicability in real-world screening contexts."

"This design has the potential to broaden the applicability of the screening tool, allowing its use by professionals from diverse fields and in varied settings, including community and primary care, thereby enhancing opportunities for early identification of individuals at risk for dysphagia and facilitating timely intervention across multiple levels of healthcare."

" individuals at risk in a more accessible and scalable manner".

"The results demonstrate diagnostic performance consistent with screening tool requirements, particularly in sensitivity."

3. Sampling and Sample Size Issues

Convenience sampling bias: While acknowledged, the impact on model generalizability is underestimated. The Discussion should include a deeper analysis of how selection bias may affect external validity.
Sample size justification: No sample size calculation is provided. Diagnostic accuracy studies require predetermined calculations based on expected sensitivity, specificity, and desired confidence intervals.
Missing data: 43 missing values were deleted without reporting missingness patterns or conducting sensitivity analyses to validate this approach.
Class distribution: Although SMOTE was applied, the class distribution between the training and test sets should be explicitly reported.

Answer: *We acknowledge that the use of convenience sampling may limit the external validity of the findings. However, the study population was intentionally composed of individuals referred for videofluoroscopy, reflecting the clinical context in which the proposed model is intended to be applied.*
In the revised manuscript, we expanded the Discussion to clarify that this sampling strategy may affect the sample's representativeness and limit direct generalization to community-based or asymptomatic populations. Nevertheless, the inclusion of a clinically referred population is consistent with diagnostic accuracy studies conducted in specialized dysphagia settings.

We also emphasize that differences in clinical spectrum and case-mix across populations may affect the stability and transportability of model performance estimates. Therefore, although the model demonstrated promising diagnostic performance in this clinical context, external validation in multicenter and more heterogeneous populations remains necessary.
In addition, a participant flowchart was added to improve transparency regarding excluded cases and missing data. The revised manuscript now provides a clearer description of the missing values identified during data preprocessing and the reasons for exclusion. We also expanded the description of class distribution across the training and test datasets, including the distribution before and after SMOTE application.

"The use of convenience sampling may limit the external validity of the findings, as participants were recruited from a specialized clinical population referred for videofluoroscopy. However, this recruitment strategy reflects the intended clinical application setting of the model. Differences in case-mix and clinical characteristics across populations may influence model transportability, reinforcing the need for external validation in multicenter and more heterogeneous samples."

We agree that formal sample size planning is important in diagnostic accuracy studies. In the present study, no a priori sample size calculation was performed because this was an exploratory study based on the available sample during the study period. Rather than relying on a post hoc power calculation, we acknowledged this as a limitation and emphasized the precision of the estimated diagnostic performance measures. The number of dysphagia cases included allowed an exploratory assessment of sensitivity and specificity; however, future validation studies should include formal sample size calculations based on expected sensitivity, specificity, and outcome prevalence.

"Given the exploratory nature of this study, no formal a priori sample size calculation was performed. The sample was defined by the number of eligible participants recruited during the study period. Although the number of confirmed dysphagia cases allowed an exploratory evaluation of diagnostic performance, future validation studies should adopt formal sample size calculations based on expected sensitivity, specificity, prevalence, and desired precision."

We agree that clearer reporting of class distribution is important. We have revised the Methods section to explicitly report:

- *The original class distribution (305 non-dysphagia vs. 150 dysphagia)*
- *The separation into training (75%) and test (25%) datasets*
- *The application of SMOTE only to the training set, resulting in balanced classes (229 vs. 229)*
- *The preservation of the original class distribution in the test set to ensure unbiased performance evaluation*

This clarification improves transparency and aligns with best practices in machine learning model evaluation.

"SMOTE was applied exclusively to the training set, while the test set maintained the original class distribution."

"Due to class imbalance, the Synthetic Minority Over-sampling Technique (SMOTE) algorithm [31] was applied to artificially increase the minority class only in the training data. During cross-validation, SMOTE was applied exclusively to the training folds, while the validation folds and the independent test set remained unchanged and contained only real instances to preserve the validity of the model evaluation.} After oversampling, the training data became balanced, with 229 instances per class, for a total of 458 samples used for model training and optimization. The independent test set maintained the original class distribution."

4. **Critical Methodological Flaw: Verification Bias**

The manuscript contains contradictory statements regarding gold standard assessment:

"Individuals who presented a clinical speech-language diagnosis of dysphagia were referred for confirmation..."

"150 (32.9%) were confirmed to have dysphagia by videofluoroscopy"

Required clarifications:

How many of the 455 participants actually underwent videofluoroscopic swallowing study (VFSS)?

Was VFSS performed only on PARD-positive cases? If so, this constitutes verification bias, artificially inflating reported sensitivity and specificity.

Provide a CONSORT-style flow diagram detailing participant enrollment, screening outcomes, and gold standard assessment.

Answer: *We thank the reviewer for highlighting the need for greater clarity regarding the reference standard assessment.*

In this study, all participants underwent clinical swallowing assessment using PARD. Videofluoroscopic swallowing study (VFSS) was performed in participants with suspected dysphagia according to the clinical assessment protocol, reflecting the institutional clinical workflow.

To improve methodological transparency, we added a STARD-based participant flow diagram detailing participant enrollment, clinical assessment, VFSS referral, exclusions, missing data, and final classification.

The participant flow diagram based on STARD recommendations is presented in Figure 2, detailing participant enrollment, clinical assessment, VFSS referral, exclusions, missing data, and final diagnostic classification.

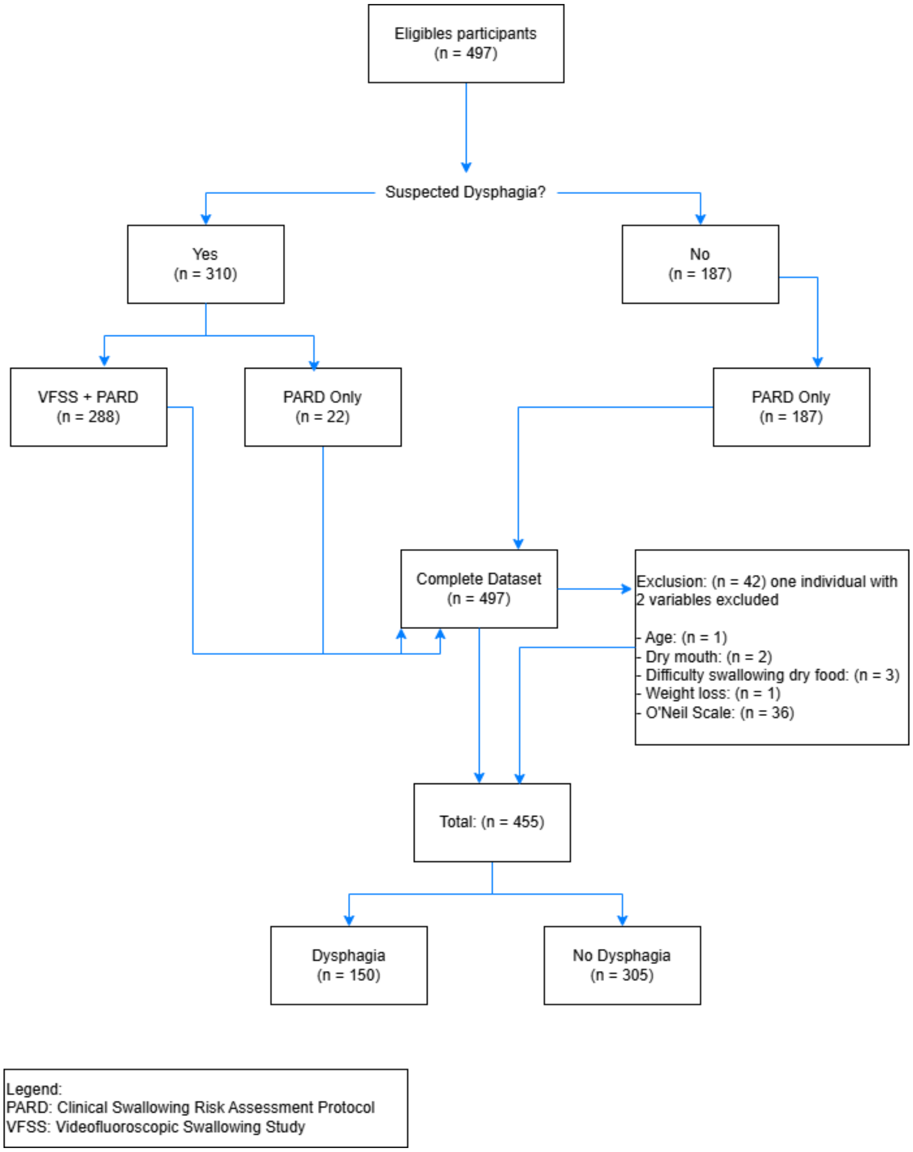

Figure 2. Participant flow diagram based on STARD recommendations

5. Machine Learning Methodology

Hyperparameter selection: Table 1 presents hyperparameters without justification (grid search, random search, or empirical selection?). The CNN architecture (single convolutional layer) is unusually simple for structured data; a rationale for this choice versus traditional approaches is needed.

Data leakage risk: The description states: "The dataset was initially split... allocating 25% for testing and 75% for training," followed by "5-fold cross-validation (CV) on the training set." Critical ambiguity remains: Was SMOTE applied before CV or within each CV fold? Application before CV creates data leakage and overly optimistic performance estimates.

Correct pipeline: Raw data → Train/test split → CV on training data only → SMOTE applied to each training fold separately → Final evaluation on untouched test set.

Answer: *We acknowledge that the description of hyperparameter selection was insufficient.*

In this study, hyperparameters were defined using a combination of empirical selection and standard values commonly reported in the literature, considering the exploratory nature of the study and the objective of comparing multiple algorithms under consistent conditions.

We have now clarified this in the Methods section, explicitly stating that:

- *No formal grid search or randomized optimization procedure was performed*
- *Hyperparameters were selected based on prior knowledge, default configurations, and preliminary testing*
- *The primary goal was to evaluate comparative model performance rather than optimize a single model*

Regarding the Convolutional Neural Network (CNN), we agree that the architecture is relatively simple. This choice was intentional, given that:

- *The dataset consists of structured tabular data, not images or sequential signals*
- *Simpler architectures reduce the risk of overfitting in moderate sample sizes*
- *The aim was to test whether even low-complexity neural models could perform competitively in this context*

We have added this rationale to the manuscript to better justify the modeling choices.

Hyperparameters were defined based on standard configurations reported in the literature and exploratory testing, without a formal grid or randomized search.

About data leakage and SMOTE application

Answer: We thank the reviewer for this important observation.
To clarify the methodology and further strengthen the validation procedure, we revised the training pipeline to ensure that data resampling is performed exclusively within each cross-validation training fold.

In the updated workflow, the dataset is first divided into independent training and test sets. Cross-validation is then conducted only on the training data, with SMOTE applied separately within each training fold, while validation folds and the final test set remain completely untouched throughout the process.

All models were retrained and reevaluated using this revised pipeline, providing a more rigorous assessment of model generalization performance.

"Due to class imbalance, the Synthetic Minority Over-sampling Technique (SMOTE) algorithm [29] was applied to artificially increase the minority class only in the training data. During cross-validation, SMOTE was applied exclusively to the training folds, while the validation folds and the independent test set remained unchanged,

containing only real instances to preserve the validity of the model evaluation. After oversampling, the training data became balanced, with 229 instances per class, for a total of 458 samples used for model training and optimization. The independent test set maintained the original class distribution."

6. **Model Selection Criteria**

The CNN was selected as the "best model" despite reduced sensitivity (0.84 vs. 0.87 for Naive Bayes and SVM). For public health screening applications, sensitivity prioritization is paramount—the cost of false negatives (missed dysphagia leading to aspiration pneumonia, malnutrition) substantially exceeds false positives. The model selection rationale should be reconsidered, or the advantages of Naive Bayes for sensitivity-prioritized screening should be discussed.

Answer: *We thank the reviewer for this important and clinically grounded observation.*

We agree that, in the context of public health screening for dysphagia, sensitivity is a critical parameter, as false negatives may lead to severe clinical consequences.

In our initial analysis, the Convolutional Neural Network (CNN) was selected as the best-performing model based on a global evaluation of multiple performance metrics, including accuracy, specificity, F1-score, and Matthews correlation coefficient (MCC), aiming to balance overall classification performance. However, we acknowledge that for screening purposes, prioritizing sensitivity is essential.

To address this point, we have revised the Discussion to:

Explicitly acknowledge the importance of sensitivity-driven model selection in screening contexts.

Discuss the potential advantages of using simpler, more interpretable models with high sensitivity, making them particularly suitable for screening applications.

We now clarify in the manuscript that model selection should be guided by the intended clinical application. In dysphagia screening scenarios, particularly from a public health perspective, sensitivity is a highly relevant parameter, since false-negative cases may delay clinical intervention and increase the risk of complications such as aspiration pneumonia, malnutrition, and increased morbidity. In this context, models with higher sensitivity may represent a preferable alternative for early-risk identification, even when their overall performance is lower.

Therefore, the choice of the most appropriate model should balance overall predictive performance with clinical priorities and the potential impact of diagnostic errors. This discussion has been incorporated into the manuscript to provide a more clinically grounded interpretation of the findings.

We thank the reviewer for this valuable suggestion, which improved the translational relevance of our findings.

7. **Interpretability Analysis**

Current LIME explanations are limited to two true-positive cases. Missing are:
False positive and false negative case analyses (essential for understanding failure modes)
Global feature importance measures (permutation importance, SHAP values) rather than solely local explanations.

Answer: *We agree that analyzing false-positive and false-negative cases is essential for understanding model failure modes. In addition, global perspective techniques are important. To increase transparency and understanding of the model's behavior, we have made the following updates in the discussion section.:*

First, we have expanded the Local Interpretability Analysis (LIME) to include representative false-positive and false-negative cases.

Additionally, to provide a global perspective, we have added Feature Importance by Permutation for the best-performing models. This model-agnostic analysis complements LIME by offering a robust view of the most influential variables, addressing the need for a global importance metric beyond local explanations.

8. **Inadequate Limitations Discussion**

Current limitations (sample size, convenience sampling) are insufficient. Required additions:
Verification bias risk (as detailed above)
Single-center limitation: All data from one Brazilian hospital; generalizability to diverse populations and healthcare systems is unknown
Temporal factors: Data collection spanned December 2021 to January 2025, encompassing COVID-19 pandemic periods that may affect variables such as COVID-19 history
Absence of external validation on independent datasets
Lack of prospective assessment in real-world screening workflows

Answer: *We thank the reviewer for this observation.*

We acknowledge that data collection occurred during a period influenced by the COVID-19 pandemic. However, in our sample, the proportion of participants reporting a history of COVID-19 infection was low, and an even smaller proportion required hospitalization, representing less than 10% of the total sample. Therefore, we believe that the impact of COVID-19–related factors on the variables included in the model and on the study findings is likely to be limited. To ensure transparency, we have added a brief statement in the Discussion acknowledging the temporal context of data collection, while clarifying that COVID-19 does not appear to have significantly influenced the sample characteristics. All other potential limitations have already been discussed.

"This study has limitations, including the sample size, since a larger sample and more significant variability of the participants' characteristics can improve the algorithm's performance. Given the exploratory nature of this study, no formal a priori sample size calculation was performed. The sample was defined by the number of eligible participants recruited during the study period. Although the number of confirmed dysphagia cases allowed an

exploratory evaluation of diagnostic performance, future validation studies should adopt formal sample size calculations based on expected sensitivity, specificity, prevalence, and desired precision. Another limitation is the convenience sample, which can select more similar individuals. The use of convenience sampling may limit the external validity of the findings, as participants were recruited from a specialized clinical population referred for videofluoroscopy. However, this recruitment strategy reflects the intended clinical application setting of the model. Differences in case-mix and clinical characteristics across populations may influence model transportability, reinforcing the need for external validation in multicenter and more heterogeneous samples. Furthermore, this was a single-center study conducted in a hospital in southern Brazil, and the applicability of the findings to other populations, healthcare systems, and epidemiological contexts remains uncertain. Data collection occurred between December 2021 and January 2025, spanning a period influenced by the COVID-19 pandemic. However, the proportion of participants reporting COVID-19 infection was low, and few required hospitalization, suggesting a limited impact on the study variables and findings.

~~In any case, this study is innovative in its proposition, using artificial intelligence to propose an algorithm capable of identifying an individual's risk of dysphagia based on 15 items of personal characteristics and basic conditions regarding general and oral health~~. In any case, this study is innovative in its approach, translating machine learning into a simplified screening model based exclusively on self-reported variables, enabling the identification of individuals at risk of dysphagia through a scalable, accessible approach applicable to non-specialized settings. Rather than introducing a novel machine learning technique, this approach emphasizes feasibility and applicability in real-world screening contexts. This study will allow professionals in different environments to identify early-on adults who need a clinical swallowing assessment by a speech therapist. The results demonstrate diagnostic performance consistent with screening tool requirements, particularly in sensitivity. ~~Their results are very good and demonstrate adequate diagnostic accuracy.”~~

9. Reporting Guidelines

Explicit statement of adherence to STARD 2015 (Diagnostic Accuracy Studies) is required—Reference 35 is cited, but guideline compliance is not declared. For machine learning components, TRIPOD-ML or CLAIM guidelines should also be referenced.

Answer: *We thank the reviewer for this important comment.*

We agree that adherence to explicit reporting guidelines improves transparency and reproducibility. In response, we have revised the manuscript to clearly state compliance with the STARD 2015 guidelines for diagnostic accuracy studies. Specifically, we have added a statement in the Methods section indicating that the study was conducted and reported in accordance with STARD 2015 recommendations. In addition, considering the machine learning components of this study, we have also incorporated references to relevant reporting frameworks, including TRIPOD-ML, to ensure appropriate reporting of model development and evaluation. These additions strengthen the methodological transparency and align the manuscript with current best practices for reporting diagnostic accuracy studies involving machine learning. Both guidelines were included as supplementary material in the article.

Reviewer #2: Methodology and Discussion: I suggest that the authors revise the manuscript to improve clarity and provide a more detailed description of the algorithms used. It is important to clearly explain how each model processes the data and what its main characteristics are, especially for those that achieved the best results (Naive Bayes, Support Vector Machine, and Convolutional Neural Network). In addition, considering that Convolutional Neural Networks are

more commonly applied to complex data such as images and videos, it would be relevant to discuss why this model showed better performance in a structured tabular clinical dataset.
A clearer explanation of the relationship between the algorithms and the type of data used would help readers better understand the methodological choices and the results obtained.

Answer: *We thank the reviewer for this valuable suggestion.*

The manuscript has been revised for clarity. In the methodology section, we create a subsection titled "Machine learning models," and explain in more detail how each model processes the data. We also highlight their main characteristics.

“Machine learning models:

The following machine learning models were evaluated: Naive Bayes (NB), K-Nearest Neighbors (KNN), Logistic Regression (LR), Decision Tree (DT), Random Forest (RF), Support Vector Machine (SVM), Multilayer Perceptron (MLP), and Convolutional Neural Networks (CNN).

NB is a probabilistic algorithm that applies Bayes' theorem under the "naive" assumption of conditional independence between attributes. The KNN is a simple and intuitive classification algorithm. KNN classifies a new data point based on the majority class of its K nearest neighbors in the training set. The algorithm is sensitive to the value of K, which indicates the number of neighbors considered. The DT constructs a hierarchical tree structure for supervised learning. Each node represents a decision based on an attribute, leading to further splits until a terminal class or value is reached. This transparent structure can handle both categorical and numerical data. LR is a statistical technique used to model the probability of a binary dependent variable. LR is used to predict a probability, which can then be transformed into a binary classification.

RF uses multiple decision trees to improve predictions. Each tree is trained on a different random subset of the data, and its results are combined by voting for classification or by averaging for regression, making it an ensemble learning technique. This approach improves accuracy and reduces error. The SVM classifies data by creating hyperplanes to separate classes. MLP consists of one or more hidden layers of neurons and an output layer. The most common architecture is fully connected, where each neuron in a given layer is connected to all neurons in the subsequent layer.

A CNN consists of convolutional and pooling layers that progressively extract and compress relevant features from the input data. As the network deepens, these representations become more complex. The extracted features are then fed into a MLP, which performs the final prediction.”

Additionally, to address your point regarding the statement that “Convolutional Neural Networks are more commonly applied to complex data such as images and videos”, we revised the Discussion section to:

Explicitly acknowledge the importance of sensitivity-driven model selection in screening contexts.

Discuss the potential advantages of simpler and more interpretable models with high sensitivity, which may be particularly suitable for screening applications.
