# Supplementary material for: Artificial intelligence for dysphagia screening: A machine learning approach
Source: PLOS Digit Health. 2026 Sep 29;5(9):e0001755. doi: 10.1371/journal.pdig.0001755 (PMC13623117; doi:10.1371/journal.pdig.0001755)
Supplement: Supplementary file 5 [file pdig.0001755.s006.pdf]

Manuscript PDIG-D-26-00067R1

Dear,
Leo Anthony Celi
Editor-in-Chief
PLOS Digital Health

We sincerely thank the reviewers for their comments and suggestions regarding the proposed changes to the scientific article. We value all comments received, as they contribute to the quality of our publication and strengthen the focus on artificial intelligence and dysphagia screening methods.

We have incorporated the reviewers' suggestions into the previously submitted manuscript and performed a comprehensive review of the material to increase clarity and coherence. The most relevant changes are highlighted in red throughout the text.

We are available for further clarification.
All authors.
----

3. Sampling, Sample Size, and Missing Data
Authors' response: The authors expanded the Discussion on convenience sampling limitations, acknowledged the lack of a priori sample size calculation, and explicitly reported class distributions before/after SMOTE and across train/test splits.
Referee comment: The reporting of class distribution and SMOTE application is now transparent. However, missing data handling still lacks rigor: the 43 excluded observations were removed without reporting missingness patterns (e.g., MCAR vs. MAR) or conducting sensitivity analyses to validate the deletion approach. Given that 36 of the 42 exclusions were due to missing O'Neill Scale data (per Figure 2), this is not a trivial issue. The authors are advised to add a brief missingness analysis.

<u>Answer:</u> *We thank the reviewer for this important comment. We revisited the missing data process and clarified its origin in the manuscript. The absence of O'Neill Scale data was linked to the timing of the analytical database's construction, when some VFSS reports had not yet been issued. Since the O'Neill Scale classification relies directly on VFSS interpretation, these participants could not be assigned a dysphagia severity score. Therefore, the missingness was procedural—driven by report-processing turnaround rather than by participant characteristics or by the unobserved O'Neill Scale value itself. This provides evidence against a Missing Not At Random (MNAR) mechanism and is most consistent with a Missing At Random (MAR) assumption. To further assess the impact of case exclusion, we also performed sensitivity analyses comparing the primary analysis with analyses restricted to participants with complete VFSS data.*

~~The resulting new file had 43 missing values. Considering the characteristics of the variables, the best~~

~~approach would be to remove these observations to ensure data quality and integrity in subsequent analysis~~. Thirteen missing values were identified across 12 participants. Eight participants were missing O'Neill Scale classification because, at the time of database construction, the corresponding VFSS resposts were not available; therefore their dysphagia severity could not be determined and they were excluded from the analysis. The remaining 5 exclusions were distributed across involuntary weight loss in the last 6 months (n=1), difficulty swallowing dry foods (n=2), and dry mouth (n=2).

4. Critical Methodological Flaw: Verification Bias ❌

Authors' response: The authors provided a STARD-based participant flow diagram (Figure 2) and stated: "Videofluoroscopy was used as the reference standard for diagnosing dysphagia... All assessments were performed with blinding between the index test and the reference standard."

Referee comment: This is the most serious unresolved issue in the revised manuscript, and paradoxically, the newly added flow diagram exposes the problem more clearly than the original text:

• Of 497 eligible participants, only 288 underwent VFSS (the reference standard).

• 209 participants (22 with suspected dysphagia but PARD-only, and 187 with no suspected dysphagia and PARD-only) did not undergo VFSS.

• Yet the final analytic dataset of 455 participants is dichotomized into 150 "Dysphagia" and 305 "No Dysphagia."

The authors never clarify how the 305 "non-dysphagia" cases were verified. If a substantial proportion were classified as negative based solely on PARD (a screening tool, not a gold standard), this constitutes severe partial verification bias. In diagnostic accuracy studies, using the screening test's negative result to define the reference-standard negative class artificially inflates both specificity and accuracy, while the true sensitivity remains unknown. This issue is unacceptable and must be fully resolved.

Answer: *We thank the reviewer for this important comment. We agree that VFSS was not applied to all participants and that this constitutes an important limitation of the study. We have therefore revised the manuscript to describe the outcome ascertainment procedure more transparently. Specifically, all participants underwent a comprehensive, standardized clinical swallowing assessment conducted by a qualified speech-language pathologist, but VFSS was performed only when there was a clinical or medical indication. Performing VFSS in participants without swallowing complaints, clinical signs, or medical indications solely for research purposes would not have been ethically justifiable, given the exposure to ionizing radiation and contrast material. Participants were classified as not having dysphagia only when there was concordant evidence across all available clinical information, including the absence of self-reported swallowing complaints, previous clinical suspicion or medical recommendation for instrumental swallowing assessment, no relevant clinical history suggestive of dysphagia or aspiration, and no signs of swallowing impairment during the standardized clinical assessment. Participants with clinical signs suggestive of dysphagia who did not undergo VFSS were excluded from the analytic sample. Because there was no clinical indication for instrumental investigation in these individuals, they were not referred for VFSS. We acknowledge that, because VFSS was not systematically applied to all participants, some misclassification of the non-dysphagia group cannot be ruled out and partial verification bias may have occurred. To assess the potential*

impact of this limitation, we performed sensitivity analyses restricted to participants who underwent VFSS and compared the diagnostic performance estimates with those obtained in the full analytic sample. These analyses quantify, but do not eliminate, the potential impact of indication-driven verification. We have revised the Abstract, Methods, Results, Discussion, and Conclusion to report these analyses, explicitly acknowledge this limitation, and caution against interpreting the performance estimates from the full sample as equivalent to those from a fully verified diagnostic accuracy study. The sensitivity analysis is described and presented in detail in Section 2, following the recommendation for a major revision. The complete analysis will be made available in the main document and supplementary material.

"Adults and older adults were screened and assessed, and a videofluoroscopy swallowing study was performed to confirm dysphagia in participants with complaints about swallowing and signs or symptoms identified in the clinical assessment by a speech therapist."

…

"A convenience sample of adults aged 18 years or older was recruited from two sources. The sample included individuals referred for videofluoroscopic swallowing assessment and volunteers from the hospital and university communities who reported no swallowing complaints. All participants were invited to undergo the same study procedures."

…

"The primary outcome was the presence of oropharyngeal dysphagia, determined using a prespecified two-stage assessment procedure. Regardless of the recruitment source, all participants underwent the same standardized clinical swallowing assessment. Participants were subsequently classified according to the presence or absence of swallowing complaints, previous clinical or medical suspicion of dysphagia, findings from the standardized clinical assessment, and, when clinically indicated, videofluoroscopic findings.

Swallowing was assessed both indirectly and directly. The indirect assessment measured the maximum number of spontaneous saliva swallows performed within one minute. The direct assessment involved the intake of foods and liquids at different consistencies, according to the International Dysphagia Diet Standardisation Initiative (IDDSI) criteria [32]: 200 mL of water (IDDSI level 0), 100 mL of a thickened strawberry-flavored juice preparation (IDDSI level 3), and half a slice of bread (IDDSI level 7). Each consistency was tested under controlled conditions, in which intake was initiated following the speech-language pathologist's instruction, and under free-intake conditions, in which participants consumed the food or liquid at their own pace.

During the clinical assessment, participants consumed at least three sips of thin liquid, four spoonfuls of the thickened preparation, and three portions of solid food. Oxygen saturation, ambient noise, and swallowing sounds were monitored throughout the assessment. Cervical auscultation was recorded before, during, and after swallowing using an Eko Core amplifier (Eko Health, United States of America) coupled to a 3M Littmann stethoscope (3M, United States of America), positioned lateral to the trachea and immediately below the cricoid cartilage. Following the assessment, the speech-language pathologist completed the Clinical Swallowing Risk Assessment Protocol (PARD) [33]. The protocol assesses variables including anterior oral leakage, oral transit time, nasal reflux, number of swallows, laryngeal elevation, cervical auscultation findings, oxygen desaturation, vocal quality, coughing, choking, and other clinical signs suggestive of swallowing impairment.

Participants recruited from the hospital or university communities who had no self-reported swallowing complaints, no previous clinical suspicion or medical indication for instrumental assessment, and no signs suggestive of dysphagia on the standardized clinical assessment were classified as not having dysphagia for the purposes of this study. Because these participants did not present any clinical indication for instrumental investigation, they were not referred for videofluoroscopic swallowing assessment.

Participants were referred for videofluoroscopy when at least one of the following conditions was present: a prior medical recommendation for instrumental swallowing assessment, a clinical suspicion of

dysphagia, signs or symptoms suggestive of swallowing impairment, or findings indicative of dysphagia on the standardized clinical assessment. This included participants who had already been referred for videofluoroscopy as part of their hospital care and participants recruited from the hospital or university communities in whom a possible swallowing impairment was identified during the study assessment. For these participants, the final outcome classification was based on the videofluoroscopic findings.

The videofluoroscopic swallowing studies were performed at the hospital's outpatient radiology department by a speech-language pathologist and a radiology technician. The speech-language pathologist responsible for the videofluoroscopic assessment was different from the speech-language pathologist who conducted the standardized clinical swallowing assessment.

During the videofluoroscopic assessment, participants received thin liquid (IDDSI level 0) through controlled and self-paced cup sips and a homogeneous thickened preparation (IDDSI level 3) administered with a dessert spoon, bread, and crackers. Barium sulfate contrast medium was added to the foods and liquids being tested. The examination was performed with the participant seated and included lateral and anteroposterior views, allowing visualization of bolus transit through the oral, pharyngeal, and esophageal phases of swallowing.

The presence and severity of oropharyngeal dysphagia were classified using the O'Neil Swallowing Scale [34], with dysphagia categorized as mild, mild-to-moderate, moderate, moderate-to-severe, or severe. Videofluoroscopy was therefore used as the reference standard for participants who met the clinical or medical criteria for instrumental swallowing assessment. For participants without swallowing complaints, previous suspicion, medical indication, or clinical signs of swallowing impairment, classification as not having dysphagia was based on the concordant absence of these findings.

The clinical and videofluoroscopic assessments were performed independently and under blinded conditions. The speech-language pathologist conducting the standardized clinical assessment was blinded to the videofluoroscopic findings. Conversely, the speech-language pathologist and physician responsible for the videofluoroscopic interpretation were blinded to the results of the standardized clinical assessment and the machine-learning model outputs. The professionals responsible for the clinical and videofluoroscopic assessments did not participate in the development or interpretation of the machine learning models.

The machine learning models were treated as index tests, and their outputs were generated independently of the clinical and videofluoroscopic classifications. When videofluoroscopy was indicated, the index-test data and the reference-standard assessment were obtained within the same clinical assessment period. Participants with incomplete data required for outcome classification or model development were excluded from the analyses."

…

"Machine learning models based exclusively on self-reported information may help identify adults more likely to require a comprehensive swallowing assessment. Because these models use variables that can be collected without specialized equipment, they may represent an accessible approach to preliminary risk stratification in outpatient and community settings. However, their results should not be interpreted as a diagnosis of dysphagia or as a substitute for a standardized clinical swallowing assessment or instrumental examination when clinically indicated. Moreover, the effects of using these models on referral pathways, access to care, demand for specialized services, and patient outcomes were not evaluated in the present study and should be investigated before their implementation in clinical practice.

This study has limitations. First, no formal a priori sample size calculation was performed due to the study's exploratory nature. The sample size was determined by the number of eligible participants recruited during the study period. Although the number of participants classified as having dysphagia enabled an exploratory assessment of model performance, future validation studies should determine the required sample size based on the expected prevalence of dysphagia, sensitivity and specificity, the number of candidate predictors, and the desired precision of the estimates. Second, convenience sampling may have introduced selection bias and limited the generalizability of the findings. Participants were recruited from two sources: adults receiving outpatient care who had been referred for videofluoroscopic swallowing assessment and volunteers from the hospital and university communities. The study did not include individuals receiving emergency care or patients with severe or medically unstable conditions. Therefore, the sample primarily represents adults assessed in outpatient and community settings and may

not reflect the full clinical spectrum of dysphagia. In particular, the performance of the models in individuals with acute illness, severe dysphagia, complex medical conditions, or a high risk of aspiration remains unknown. The absence of these populations may also have reduced the variability and severity of dysphagia represented in the sample.

Another important limitation concerns outcome ascertainment. VFSS was not performed on all participants. Individuals with a previous medical recommendation for instrumental swallowing assessment, a clinical suspicion of dysphagia, signs or symptoms suggestive of swallowing impairment, or abnormal findings on the standardized clinical swallowing assessment were referred for VFSS. Conversely, participants without self-reported swallowing complaints, previous clinical suspicion or medical indication, and signs of dysphagia on the standardized clinical assessment were classified as not having dysphagia without undergoing VFSS. Thus, dysphagia status was determined through a two-stage procedure, and the same reference-standard assessment was not applied to the entire sample. To reduce the risk of outcome misclassification among participants who did not undergo VFSS, the assessment considered their known health conditions and clinical history, including previous pneumonia, unexplained weight loss, medical conditions associated with swallowing impairment, and other potential indicators of dysphagia or aspiration. This information was analyzed together with the absence of self-reported swallowing complaints and the absence of abnormal findings on the standardized clinical swallowing assessment. The concordant absence of complaints, relevant clinical indicators, and signs of swallowing impairment reduced the likelihood of clinically unrecognized dysphagia in these participants. Nevertheless, silent aspiration cannot be definitively excluded without instrumental assessment. Therefore, some participants with mild, subclinical, or silent swallowing impairment may have been incorrectly classified as not having dysphagia. Although the clinical assessment and review of relevant health indicators were intended to minimize this possibility, they do not eliminate the potential for partial or differential verification bias. The reported model performance estimates should therefore be interpreted with appropriate caution.

The study was conducted at a single hospital in southern Brazil, which further limits the applicability of the findings to populations with different clinical, sociodemographic, healthcare, and epidemiological characteristics. Data collection occurred between December 2021 and January 2025, a period partially influenced by the COVID-19 pandemic. Although the proportion of participants reporting COVID-19 infection was low and few required hospitalization, the possibility that the pandemic influenced recruitment patterns or the characteristics of the study population cannot be entirely excluded.

Despite these limitations, the study has relevant strengths. All participants underwent the same standardized clinical swallowing assessment based on a validated protocol. When videofluoroscopy was clinically indicated, the clinical and instrumental assessments were conducted independently by different speech-language pathologists. The speech-language pathologist responsible for the clinical assessment was blinded to the videofluoroscopic findings. Conversely, the speech-language pathologist and physician responsible for the videofluoroscopic assessment and interpretation were blinded to the clinical classification and machine learning outputs. The professionals responsible for the clinical and videofluoroscopic assessments did not participate in the development or interpretation of the machine learning models.

Another strength was the incorporation of interpretability analyses into the machine learning models. In addition to evaluating predictive performance, local explanation plots and permutation feature importance analyses were used to examine the contribution of individual variables to model predictions. Difficulty swallowing dry foods, neurodegenerative disease, age, history of stroke, history of cancer, and oral health conditions consistently contributed to predictions across different models. Local explanation plots also demonstrated how combinations of variables increased or decreased the predicted probability of dysphagia in correctly classified, false-positive, and false-negative cases. These analyses improved model transparency and enabled examination of the clinical plausibility of the predictors. Nevertheless, interpretability does not establish clinical validity or eliminate the need for external validation [29,36].

The contribution of this study lies in the exploratory development of machine learning models based exclusively on self-reported variables to identify adults who may require further swallowing assessment. The proposed approach does not introduce a novel machine learning technique and should not yet be considered ready for clinical implementation. Rather, it provides preliminary evidence regarding the feasibility of using readily obtainable information to support risk stratification and indicate the need for subsequent assessment by a speech-language pathologist.

In conclusion, machine learning models based on self-reported variables showed potential to identify adults in outpatient and community settings who may warrant further clinical swallowing assessment. However, the findings should be considered exploratory because of the convenience sample,

the exclusion of hospitalized and emergency-care patients, the single-center design, and the two-stage outcome ascertainment procedure in which videofluoroscopy was not performed in all participants. Although a standardized clinical assessment and a review of relevant health conditions and clinical indicators were used to reduce the risk of outcome misclassification, silent aspiration could not be completely excluded among participants who did not undergo instrumental assessment. These models should not replace clinical or instrumental swallowing assessments. Prospective, multicenter studies involving larger, more heterogeneous samples, including participants across varying levels of dysphagia severity and healthcare settings, are required to externally validate the models before their clinical or population-level implementation."

5. Machine Learning Methodology ⚠️

Authors' response: The authors stated that hyperparameters were selected based on literature defaults and exploratory experiments (without formal grid/random search), justified the simple CNN architecture for tabular data, and claimed to have fixed the SMOTE-CV pipeline so that SMOTE is applied exclusively within each training fold.

Referee comment: While the methodological descriptions are now clearer, the revised results differ so dramatically from the original manuscript that they raise serious concerns about the integrity of the original analysis:

Model	Original Test Sensitivity	Revised Test Sensitivity
Naive Bayes	0.87	0.74
SVM	0.87	0.76
CNN	0.84	0.76
Random Forest	0.58	0.71

Such large shifts, particularly the collapse of NB and SVM performance, and the simultaneous improvement of RF, cannot be explained solely by correcting the SMOTE-CV sequence. They strongly suggest that the original manuscript contained additional data leakage, coding errors, or analytical mistakes that have now been (partially) corrected. Furthermore, the hyperparameters themselves have changed (e.g., LR: C from 1 to 0.01, max_iter from 200 to 1000; MLP: max_iter from 300 to 1000). The authors do not explain why these changes were necessary alongside the pipeline fix.

Serious doubt must be raised. The authors should provide a detailed explanation and revision, and must release the complete analysis code (Python scripts) to the journal for independent verification.

Answer: We thank the reviewer for this important observation. We would like to clarify that, in our previous response, we did not intend to attribute the differences between the original and revised results exclusively to correcting the SMOTE cross-validation procedure. The revised analysis involved a complete rerunning of the computational workflow. In addition to ensuring that SMOTE was applied exclusively within the training portion of each cross-validation fold, the revised workflow also included a review and standardization of model-specific configurations and their consistent implementation across cross-validation, final model training, and test-set evaluation. Following the reviewer's current comment, we conducted an additional audit comparing the original and revised computational workflows. This review confirmed that the differences between the two analyses involved not only the placement of SMOTE but also changes in model-specific implementation and configuration. These differences, therefore,

contributed to the observed changes in model performance and ranking between the original and revised analyses. Specifically, the revised workflow ensured consistency between the model configurations evaluated during cross-validation and those used for final model training and test-set evaluation. Model-specific parameters were also reviewed during the complete reanalysis. The increase in the maximum number of iterations for LR and MLP was intended to ensure sufficient iterations for model convergence, while other model-specific configurations were revised and applied consistently throughout the analytical workflow. These changes were implemented as part of the methodological review of the computational pipeline and were not based on the performance of the independent test set. We acknowledge that these modifications were not sufficiently itemized in our previous response, which may have created the impression that the performance differences were attributable solely to correcting the SMOTE cross-validation procedure. Following the major revision, all machine learning models were retrained from scratch using the revised analytical dataset. Consequently, hyperparameter optimization was repeated. A small grid search was performed over the main hyperparameters of each classifier, using predefined candidate values drawn from the literature and common practice. For example, the KNN classifier was evaluated with k = [1,3,5,7], while other classifiers were optimized over a limited set of relevant hyperparameter values. The final hyperparameters reported in Table 1 correspond to the best-performing configuration obtained during this optimization process using the revised training data. Therefore, some hyperparameter values differ from those reported in the previous version. The complete analysis code has been included with the revised submission for the reviewers' audit.

6. Model Selection Criteria
Authors' response: The authors revised the Discussion to acknowledge that sensitivity should be prioritized in screening contexts. They now highlight the KNN model (sensitivity 0.79) as a viable option despite its lower overall metrics, and discuss the trade-off between sensitivity and specificity.
Referee comment: The response is conceptually appropriate. However, the revised maximum sensitivity of 0.79 (KNN) is notably lower than the originally claimed 0.87. The authors should more cautiously qualify the statement that the results demonstrate "adequate diagnostic accuracy," as a sensitivity of 0.79 for a screening tool, while better than RF's 0.71, still implies that 21% of true dysphagia cases would be missed. In a public health screening context, the wording should be more cautious and restrained.

<u>Answer:</u> We appreciate the reviewer's observation. We agree that, following revision, the highest sensitivity warrants a more cautious interpretation regarding screening performance. Accordingly, we revised the Discussion and Conclusion to replace the statement that the models demonstrated "adequate diagnostic accuracy" with more restrained language.

8. Inadequate Limitations Discussion
Authors' response: Added discussion of single-center design, COVID-19 temporal factors, lack of external validation, and need for prospective assessment.
Referee comment: These additions are welcome. However, the most fundamental limitation: verification bias (Point 4 above), remains entirely unmentioned and should be emphasized. The

authors cannot claim methodological transparency while omitting discussion of the fact that a large proportion of "non-dysphagia" cases were never verified by the reference standard. This omission must be corrected.

Answer: *We thank the reviewer for emphasizing this important limitation. We agree that the previous version of the manuscript did not adequately address the potential for verification bias arising from the fact that VFSS was not performed in all participants. In the revised manuscript, we now explicitly state that outcome ascertainment followed a two-stage procedure. Participants with a previous medical recommendation for instrumental assessment, clinical suspicion of dysphagia, swallowing-related signs or symptoms, or abnormal findings on the standardized clinical swallowing assessment were referred for VFSS. Participants without swallowing complaints, relevant clinical history, medical indication, or abnormal findings on the comprehensive clinical assessment were classified as not having dysphagia without undergoing VFSS. Therefore, the instrumental reference standard was not systematically applied to the entire sample. We also clarified that the 22 participants who were classified as having dysphagia on the clinical assessment but did not undergo VFSS were excluded from the analytic sample because their positive classification could not be instrumentally confirmed. We acknowledge that this procedure may have introduced partial or differential verification bias, particularly because some participants classified as not having dysphagia were not verified using the same reference standard applied to participants with clinical suspicion. Although the classification of these participants was based on the concordant absence of self-reported swallowing complaints, previous clinical suspicion, relevant health indicators, history of pneumonia or unexplained weight loss, and signs of swallowing impairment during a comprehensive assessment conducted with a validated protocol, mild, subclinical, or silent swallowing impairment cannot be definitively excluded without instrumental assessment. This limitation is now explicitly emphasized in the Discussion. We also revised the interpretation of the model performance estimates to indicate that they should be considered exploratory and interpreted with caution. In addition, we removed statements that could imply definitive diagnostic validity or immediate clinical applicability.*

New Concerns Arising from the Revision

1. Performance inconsistency: The dramatic decline in model performance after pipeline correction (Point 5) undermines confidence in the original findings and requires code-level verification.
2. Flow diagram logic: The STARD flow diagram (Figure 2) inappropriately includes participants who never underwent the reference standard (VFSS) in a diagnostic accuracy "Complete Dataset." This is methodologically incorrect and must be either redrawn with separate pathways for VFSS-verified vs. non-verified participants, or accompanied by explicit statistical handling of the verification bias.

Answer: *All corrections have been incorporated into the new version of the manuscript.*

Final Recommendation
Major Revision

While the authors have made genuine efforts to improve transparency and reporting, the revised manuscript fails to resolve its most critical methodological flaw: the verification bias arising from the fact that a substantial proportion of participants classified as "non-dysphagia" never underwent the reference standard (VFSS). The STARD flow diagram, intended to clarify the study workflow, instead makes this problem visually obvious. Additionally, the drastic change in model performance after analytical corrections raises serious questions about the reliability of the original results that the authors have not adequately explained.

To proceed, the authors must:

1. Clarify exactly how many of the 455 analyzed participants underwent VFSS, and how the remaining participants were classified.

<u>Answer:</u> *We thank the revisor for highlighting these important issues. We have now revised the manuscript to explicitly distinguish participants who underwent VFSS from those classified through the prespecified two-stage clinical procedure without VFSS. In the revised sample, 290 participants underwent VFSS, whereas 187 were classified as non-dysphagia based on the standardized clinical assessment and absence of clinical indication for VFSS. We also added a detailed description in the Methods (and previous answer numbers 3 and 4). Also, we added Figure 2.*

2. If partial verification exists, provide a sensitivity analysis using only VFSS-completed participants and add an explicit discussion of verification bias to the Limitations.

<u>Answer:</u> *We thank the Reviewer for this valuable suggestion. We conducted a comprehensive sensitivity analysis to assess the robustness of the proposed models under varying methodological assumptions. Specifically, four scenarios were investigated:*

Full cohort (n = 477; VFSS + PARD) with SMOTE (primary analysis);

Model	Accuracy	Sensitivity	Specificity	Precision	F1-score	MCC	ROC AUC	PR AUC (AP)
Logistic Regression	0.7863	0.8158	0.7722	0.6327	0.7126	0.5581	0.8431	0.7152
SVM	0.7778	0.7895	0.7722	0.6250	0.6977	0.5347	0.8318	0.6764
Naive Bayes	0.7607	0.8421	0.7215	0.5926	0.6957	0.5295	0.8374	0.6410
CNN	0.7778	0.7368	0.7975	0.6364	0.6829	0.5165	0.8068	0.6740
Random Forest	0.7436	0.6316	0.7975	0.6000	0.6154	0.4236	0.8135	0.6315
KNN	0.7265	0.7105	0.7342	0.5625	0.6279	0.4234	0.8005	0.6528
Decision Tree	0.7179	0.6316	0.7595	0.5581	0.5926	0.3799	0.7450	0.5324
MLP	0.7179	0.5789	0.7848	0.5641	0.5714	0.3614	0.7525	0.6370

Full cohort (n = 477; VFSS + PARD) without SMOTE;

Model	Accuracy	Sensitivity	Specificity	Precision	F1-score	MCC	ROC AUC	PR AUC (AP)
CNN	0.8034	0.7105	0.8481	0.6923	0.7013	0.5549	0.8171	0.6809
Logistic Regression	0.7949	0.6842	0.8481	0.6842	0.6842	0.5323	0.8418	0.7079
SVM	0.7863	0.6842	0.8354	0.6667	0.6753	0.5162	0.8371	0.7213
Naive Bayes	0.7607	0.7632	0.7595	0.6042	0.6744	0.4976	0.8358	0.6412
Decision Tree	0.7436	0.7368	0.7468	0.5833	0.6512	0.4605	0.7836	0.6029
KNN	0.7692	0.5789	0.8608	0.6667	0.6197	0.4576	0.8035	0.6367
MLP	0.7436	0.6053	0.8101	0.6053	0.6053	0.4154	0.7338	0.5961
Random Forest	0.7350	0.5789	0.8101	0.5946	0.5867	0.3918	0.8314	0.6495

VFSS-confirmed subset only (n = 290) with SMOTE;

Model	Accuracy	Sensitivity	Specificity	Precision	F1-score	MCC	ROC AUC	PR AUC (AP)
Decision Tree	0.7324	0.7895	0.6667	0.7317	0.7595	0.4606	0.7520	0.7248
Logistic Regression	0.6620	0.6316	0.6970	0.7059	0.6667	0.3280	0.7065	0.7700
KNN	0.6479	0.6316	0.6667	0.6857	0.6575	0.2975	0.6711	0.6851
CNN	0.6479	0.6316	0.6667	0.6857	0.6575	0.2975	0.6962	0.6715
SVM	0.6338	0.5526	0.7273	0.7000	0.6176	0.2826	0.6954	0.7572
Random Forest	0.6197	0.6579	0.5758	0.6410	0.6494	0.2342	0.7109	0.7786
Naive Bayes	0.6056	0.6053	0.6061	0.6389	0.6216	0.2108	0.6451	0.7179
MLP	0.5070	0.5526	0.4545	0.5385	0.5455	0.0072	0.6340	0.7353

VFSS-confirmed subset only (n = 290) without SMOTE.

Model	Accuracy	Sensitivity	Specificity	Precision	F1-score	MCC	ROC AUC	PR AUC (AP)
Logistic Regression	0.6761	0.6579	0.6970	0.7143	0.6849	0.3540	0.7081	0.7797
Decision Tree	0.6620	0.6316	0.6970	0.7059	0.6667	0.3280	0.7281	0.7220
KNN	0.6338	0.6842	0.5758	0.6500	0.6667	0.2614	0.6575	0.6827
SVM	0.6197	0.5526	0.6970	0.6774	0.6087	0.2510	0.6946	0.7574
CNN	0.6197	0.8684	0.3333	0.6000	0.7097	0.2408	0.7010	0.7723
Naive Bayes	0.6197	0.6316	0.6061	0.6486	0.6400	0.2373	0.6555	0.7336
Random Forest	0.6197	0.6579	0.5758	0.6410	0.6494	0.2342	0.6962	0.7510
MLP	0.5352	0.5263	0.5455	0.5714	0.5479	0.0716	0.6507	0.7459

The analyses demonstrated that restricting the evaluation to VFSS-confirmed participants reduced the overall performance of all models, as expected given the substantially smaller sample size. In contrast, using SMOTE had a comparatively smaller impact on model performance. Among the evaluated algorithms, Logistic Regression showed the most consistent performance across all scenarios, whereas CNN and Naïve Bayes exhibited greater variability depending on the dataset and balancing strategy. Although absolute performance metrics changed across the sensitivity analyses, the overall interpretation of the results remained consistent, indicating that the primary conclusions were not materially altered. We added a new subsection describing the sensitivity analysis to the Methods section, incorporated the corresponding results into the Results section (Table 8), and discussed their implications in the Discussion.

3. Explain the dramatic performance shifts and provide analysis code for independent verification. Without these clarifications, the diagnostic accuracy claims in this manuscript remain unsubstantiated.

<u>Answer</u>: We would like to clarify that the differences between the original and revised results were not solely due to correcting the SMOTE cross-validation procedure. Following the major revision, we reran the entire computational workflow from scratch. In addition to restricting SMOTE to the training portion of each cross-validation fold, we reviewed and standardized model-specific configurations to ensure consistency across cross-validation, final model training, and test-set evaluation. A subsequent audit confirmed that changes in both the SMOTE implementation and model configurations contributed to the observed differences in model performance and ranking. Because all models were retrained using the revised analytical dataset, hyperparameter optimization was also repeated using predefined, literature-based search spaces. Consequently, some hyperparameter values differ from those reported in the previous version. The complete analysis code has been included with the revised submission for the reviewers' audit.
