# Supplementary material for: Artificial intelligence for dysphagia screening: A machine learning approach
Source: PLOS Digit Health. 2026 Sep 29;5(9):e0001755. doi: 10.1371/journal.pdig.0001755 (PMC13623117; doi:10.1371/journal.pdig.0001755)
Supplement: Supplementary file 6 [file pdig.0001755.s004.docx]

**Referee Report for PLOS Digital Health**

Manuscript ID: PDIG-D-26-00067

Title: Artificial Intelligence for Dysphagia Screening: A Machine Learning Approach

Referee: Huan Du, Chief Physician

Affiliation: Department of Rehabilitation Medicine, Suzhou Ninth People's Hospital

E-mail: xykk@qq.com

Date: February 14, 2026

Recommendation: Major Revision

General Comments

This manuscript presents a diagnostic accuracy study evaluating machine learning algorithms for dysphagia screening using 15 self-reported variables. While the topic is clinically relevant and the sample size (n=455) is adequate for exploratory analysis, several critical methodological and ethical issues require substantial revision before consideration for publication.

Specific Comments

1. Critical Issue: Fictitious or AI-Generated Citation

Reference 32 contains severe inaccuracies that suggest either fabrication or excessive reliance on AI-generated content without verification.

Manuscript citation:

Malandraki GA, Markaki V, Georgopoulos VC, Kapsalakis G, et al. The predictive dysphagia score (PreDyScore) in the short- and medium-term post-stroke: a putative tool in PEG indication. Eur J Phys Rehabil Med. 2016;52(2):207–216.

Verified correct citation:

Gandolfo C, Sukkar S; on the behalf of the PreDyScore Group; Ceravolo MG, Cortinovis F, Finocchi C, Gradaschi R, Orlandoni P, Reale N, Ricci S, Vassallo D, Zini A. The predictive dysphagia score (PreDyScore) in the short- and medium-term post-stroke: a putative tool in PEG indication. Neurol Sci. 2019 Aug;40(8):1619-1626. doi: 10.1007/s10072-019-03896-2. PMID: 31004231.

Required actions:

Authors must provide documentation of how this erroneous citation was generated

Complete audit of all references to verify accuracy

Explanation in the response letter regarding this discrepancy

2. Overstated Innovation Claim

The manuscript claims to be "innovative in its approach"; however, similar studies using machine learning for dysphagia prediction have been published (e.g., Jauk et al., 2023; Park et al., 2023). The contribution should be reframed as a community-based screening tool using self-reported data rather than a novel methodological approach. This distinction is important for accurate positioning within the existing literature.

3. Sampling and Sample Size Issues

Convenience sampling bias: While acknowledged, the impact on model generalizability is underestimated. The Discussion should include deeper analysis of how selection bias may affect external validity.

Sample size justification: No sample size calculation is provided. Diagnostic accuracy studies require predetermined calculations based on expected sensitivity, specificity, and desired confidence intervals.

Missing data: 43 missing values were deleted without reporting missingness patterns or conducting sensitivity analyses to validate this approach.

Class distribution: Although SMOTE was applied, the class distribution in training versus test sets should be explicitly reported.

4. Critical Methodological Flaw: Verification Bias

The manuscript contains contradictory statements regarding gold standard assessment:

"Individuals who presented a clinical speech-language diagnosis of dysphagia were referred for confirmation..."

"150 (32.9%) were confirmed to have dysphagia by videofluoroscopy"

Required clarifications:

How many of the 455 participants actually underwent videofluoroscopic swallowing study (VFSS)?

Was VFSS performed only on PARD-positive cases? If so, this constitutes verification bias, artificially inflating reported sensitivity and specificity.

Provide a CONSORT-style flow diagram detailing participant enrollment, screening outcomes, and gold standard assessment.

5. Machine Learning Methodology

Hyperparameter selection: Table 1 presents hyperparameters without justification (grid search, random search, or empirical selection?). The CNN architecture (single convolutional layer) is unusually simple for structured data; rationale for this choice versus traditional approaches is needed.

Data leakage risk: The description states: "The dataset was initially split... allocating 25% for testing and 75% for training," followed by "5-fold cross-validation (CV) on the training set." Critical ambiguity remains: Was SMOTE applied before CV or within each CV fold? Application before CV creates data leakage and overly optimistic performance estimates.

Correct pipeline: Raw data → Train/test split → CV on training data only → SMOTE applied to each training fold separately → Final evaluation on untouched test set.

6. Model Selection Criteria

The CNN was selected as the "best model" despite reduced sensitivity (0.84 vs. 0.87 for Naive Bayes and SVM). For public health screening applications, sensitivity prioritization is paramount—the cost of false negatives (missed dysphagia leading to aspiration pneumonia, malnutrition) substantially exceeds false positives. The model selection rationale should be reconsidered, or the advantages of Naive Bayes for sensitivity-prioritized screening should be discussed.

7. Interpretability Analysis

Current LIME explanations are limited to two true-positive cases. Missing are:

False positive and false negative case analyses (essential for understanding failure modes)

Global feature importance measures (permutation importance, SHAP values) rather than solely local explanations

8. Inadequate Limitations Discussion

Current limitations (sample size, convenience sampling) are insufficient. Required additions:

Verification bias risk (as detailed above)

Single-center limitation: All data from one Brazilian hospital; generalizability to diverse populations and healthcare systems is unknown

Temporal factors: Data collection spanned December 2021 to January 2025, encompassing COVID-19 pandemic periods that may affect variables such as COVID-19 history

Absence of external validation on independent datasets

Lack of prospective assessment in real-world screening workflows

9. Reporting Guidelines

Explicit statement of adherence to STARD 2015 (Diagnostic Accuracy Studies) is required—Reference 35 is cited but guideline compliance is not declared. For machine learning components, TRIPOD-ML or CLAIM guidelines should also be referenced.

Confidential Remarks to the Editor

The citation error in Reference 32 is highly concerning. The complete mismatch in authors, journal, year, and volume suggests either deliberate fabrication or unverified AI-generated content. This requires thorough investigation before any revision can be considered. Additionally, the potential verification bias in gold standard assessment represents a fundamental threat to the study's validity that must be addressed with transparent reporting of the actual VFSS completion rate.

Huan Du

Chief Physician

Department of Rehabilitation Medicine

Suzhou Ninth People's Hospital

E-mail: xykk@qq.com
