# Supplementary material for: Artificial intelligence for dysphagia screening: A machine learning approach
Source: PLOS Digit Health. 2026 Sep 29;5(9):e0001755. doi: 10.1371/journal.pdig.0001755 (PMC13623117; doi:10.1371/journal.pdig.0001755)
Supplement: Supplementary file 7 [file pdig.0001755.s007.docx]

**Reviewer’s Decision Letter**

**To the Editor, *PLOS Digital Health***

**Manuscript ID**: PDIG-D-26-00067R2

**Title**: Artificial Intelligence for Dysphagia Screening: A Machine Learning Approach

**Decision**: Minor Revision

**Overall Assessment**

The authors have made a substantial and commendable effort to address the concerns raised in the previous review round. The R2 revision demonstrates genuine engagement with the most critical issues, particularly the verification bias concern and the unexplained performance shifts between analyses.

The revised manuscript now includes: (1) a transparent description of the two-stage outcome ascertainment procedure, with explicit distinction between VFSS-verified and clinically-classified participants; (2) a comprehensive sensitivity analysis restricted to the VFSS-confirmed cohort (n=290), with results reported across multiple algorithms and both with and without SMOTE; (3) an expanded Limitations section that explicitly acknowledges verification bias as a fundamental limitation; and (4) an offer to release the complete analysis code for independent verification.

The authors' response to the performance shift concern is credible: the R2 analysis represents a complete rerun of the computational workflow, involving not only the SMOTE-CV correction but also a systematic review and standardization of model-specific configurations. The provision of analysis code for audit is the appropriate remedy.

One issue remains that requires attention before acceptance: the verification bias, while now transparently disclosed and subjected to sensitivity analysis, still affects the interpretability of the primary performance estimates. The authors should provide a clearer, quantified discussion of how the sensitivity analysis results differ from the primary analysis, and what this implies for the clinical interpretation of the model performance. This can be addressed through textual revision without additional experiments.

I therefore recommend **minor revision**.

**Comments for the Authors**

**1. Verification bias – provide a more quantified comparison between primary and sensitivity analyses**

The authors have done an excellent job in acknowledging and analyzing verification bias. The sensitivity analysis restricted to the 290 VFSS-confirmed participants is a rigorous addition. However, the Discussion currently presents the sensitivity analysis results in qualitative terms ("overall performance... was lower than that observed in the primary analysis, consistent with the substantially smaller sample size"). This does not fully convey the clinical implications of the bias.

**Recommendation**: Add a brief, quantified statement comparing the key performance metrics (e.g., sensitivity, specificity, accuracy, MCC) between the primary analysis (n=477) and the VFSS-only analysis (n=290) for the best-performing model(s) – preferably Logistic Regression or Naive Bayes. This will help readers understand the magnitude of potential overestimation due to verification bias. For example: "For Logistic Regression, sensitivity decreased from 0.82 in the primary analysis to 0.63 in the VFSS-confirmed cohort, while specificity decreased from 0.77 to 0.69." Such a comparison would provide a more transparent assessment of the bias's impact.

**2. Performance shift – confirm code availability for review**

The authors have stated that "the complete analysis code has been included with the revised submission for the reviewers' audit." This is the appropriate response to the concern about performance inconsistency. Please confirm that the code is indeed accessible and that it reproduces the reported results. If not already done, the authors should ensure the code is well-documented enough for an independent reviewer to verify the analytical pipeline.

**Recommendation**: The authors should confirm in their response that the code is fully reproducible and, if possible, provide a brief README or instructions for running the analysis. This is not a requirement for publication but would significantly strengthen transparency.

**3. Missing data – minor clarification on exclusion rationale**

The authors have explained that missing O'Neill Scale data were procedural (VFSS reports not yet issued at the time of dataset compilation) and most consistent with MAR. This is a reasonable justification. However, the manuscript states that "the best approach is to exclude these observations" without elaborating on why multiple imputation or other missing data methods were not considered.

**Recommendation**: Add a brief sentence explaining why listwise deletion was preferred (e.g., small proportion of missing data, or the MAR assumption being reasonable given the procedural nature of missingness). This is a minor clarification.

**4. Wording of conclusions – maintain the cautious tone**

The authors have appropriately toned down the claims in the Discussion and Conclusion. I appreciate the revised language: "These models should not replace clinical or instrumental swallowing assessments." and "the findings should be considered exploratory." Please ensure this cautious tone is maintained throughout, particularly in the Abstract where the phrase "adequate diagnostic accuracy" still appears.

**Recommendation**: In the Abstract, consider revising "demonstrating adequate diagnostic accuracy" to "demonstrating exploratory diagnostic accuracy that warrants further validation" or similar phrasing to maintain consistency with the Discussion's cautious framing.

**Conclusion**

The authors have responded thoroughly to the previous review concerns. The verification bias is now transparently disclosed and analyzed; the performance shift is explained and accompanied by code availability; the limitations discussion is substantially expanded; and the framing is appropriately cautious. The remaining issues are minor and can be addressed through textual clarification without additional experiments. I recommend acceptance after **minor revisions**.

Sincerely,

Reviewer
