# Supplementary material for: Artificial intelligence for dysphagia screening: A machine learning approach
Source: PLOS Digit Health. 2026 Sep 29;5(9):e0001755. doi: 10.1371/journal.pdig.0001755 (PMC13623117; doi:10.1371/journal.pdig.0001755)
Supplement: Peer review history

# Peer review: Decision Letter 0

**DOI:** 10.1371/journal.pdig.0001755.r001

**Authors:** Saskia Haitjema, Leo Anthony Celi

19 Mar 2026

Response to Reviewers Revised Manuscript with Track Changes Manuscript **Journal Requirements:**

1. Please amend your detailed Financial Disclosure statement. This is published with the article. It must therefore be completed in full sentences and contain the exact wording you wish to be published.

i. State the initials, alongside each funding source, of each author to receive each grant. For example: "This work was supported by the National Institutes of Health (####### to AM; ###### to CJ) and the National Science Foundation (###### to AM)."

2. Please ensure that your Ethics Statement is available in its entirety at the beginning of your Methods section, under a subheading 'Ethics Statement'. It must include:

i) The name(s) of the Institutional Review Board(s) or Ethics Committee(s)

ii) The approval number(s), or a statement that approval was granted by the named board(s)

iii) (for human participants or donors) - A statement that formal consent was obtained (must state whether verbal/written) OR the reason consent was not obtained (e.g., anonymity).

3. We ask that a manuscript source file is provided at Revision. Please upload your manuscript file as a .doc, .docx, .rtf or .tex.

4. Please upload separate figure files in .tif or .eps format. Also, remove the figures from your manuscript file but keep the legends.

For more information about figure files please see our guidelines:

https://journals.plos.org/digitalhealth/s/figures

https://journals.plos.org/digitalhealth/s/figures#loc-file-requirements

5. Please provide an Author Summary. This should appear in your manuscript between the Abstract (if applicable) and the Introduction, and should be 150–200 words long. The aim should be to make your findings accessible to a wide audience that includes both scientists and non-scientists. Sample summaries can be found on our website under Submission Guidelines:

https://journals.plos.org/digitalhealth/s/submission-guidelines#loc-parts-of-a-submission

6. When completing the data availability statement of the submission form, you indicated that you will make your data available on acceptance. We strongly recommend all authors decide on a data sharing plan before acceptance, as the process can be lengthy and hold up publication timelines. Please note that, though access restrictions are acceptable now, your entire data will need to be made freely accessible if your manuscript is accepted for publication. This policy applies to all data except where public deposition would breach compliance with the protocol approved by your research ethics board. If you are unable to adhere to our open data policy, please kindly revise your statement to explain your reasoning and we will seek the editor's input on an exemption. Please be assured that, once you have provided your new statement, the assessment of your exemption will not hold up the peer review process.

**Additional Editor Comments (if provided):** **Reviewers' Comments:**

**Comments to the Author**

1. Does this manuscript meet PLOS Digital Health’s publication criteria?

Reviewer #1: No

Reviewer #2: Yes

**********

2. Has the statistical analysis been performed appropriately and rigorously?

Reviewer #1: No

Reviewer #2: Yes

**********

3. Have the authors made all data underlying the findings in their manuscript fully available (please refer to the Data Availability Statement at the start of the manuscript PDF file)?

The PLOS Data policy

Reviewer #1: Yes

Reviewer #2: Yes

**********

4. Is the manuscript presented in an intelligible fashion and written in standard English?

Reviewer #1: Yes

Reviewer #2: Yes

**********

**Reviewer #1:**  General Comments

This manuscript presents a diagnostic accuracy study evaluating machine learning algorithms for dysphagia screening using 15 self-reported variables. While the topic is clinically relevant and the sample size (n=455) is adequate for exploratory analysis, several critical methodological and ethical issues require substantial revision before consideration for publication.

Specific Comments

1. Critical Issue: Fictitious or AI-Generated Citation

Reference 32 contains severe inaccuracies that suggest either fabrication or excessive reliance on AI-generated content without verification.

Manuscript citation:

Malandraki GA, Markaki V, Georgopoulos VC, Kapsalakis G, et al. The predictive dysphagia score (PreDyScore) in the short- and medium-term post-stroke: a putative tool in PEG indication. Eur J Phys Rehabil Med. 2016;52(2):207–216.

Verified correct citation:

Gandolfo C, Sukkar S; on the behalf of the PreDyScore Group; Ceravolo MG, Cortinovis F, Finocchi C, Gradaschi R, Orlandoni P, Reale N, Ricci S, Vassallo D, Zini A. The predictive dysphagia score (PreDyScore) in the short- and medium-term post-stroke: a putative tool in PEG indication. Neurol Sci. 2019 Aug;40(8):1619-1626. doi: 10.1007/s10072-019-03896-2. PMID: 31004231.

Required actions:

Authors must provide documentation of how this erroneous citation was generated

Complete audit of all references to verify accuracy

Explanation in the response letter regarding this discrepancy

2. Overstated Innovation Claim

The manuscript claims to be "innovative in its approach"; however, similar studies using machine learning for dysphagia prediction have been published (e.g., Jauk et al., 2023; Park et al., 2023). The contribution should be reframed as a community-based screening tool using self-reported data rather than a novel methodological approach. This distinction is important for accurate positioning within the existing literature.

3. Sampling and Sample Size Issues

Convenience sampling bias: While acknowledged, the impact on model generalizability is underestimated. The Discussion should include deeper analysis of how selection bias may affect external validity.

Sample size justification: No sample size calculation is provided. Diagnostic accuracy studies require predetermined calculations based on expected sensitivity, specificity, and desired confidence intervals.

Missing data: 43 missing values were deleted without reporting missingness patterns or conducting sensitivity analyses to validate this approach.

Class distribution: Although SMOTE was applied, the class distribution in training versus test sets should be explicitly reported.

4. Critical Methodological Flaw: Verification Bias

The manuscript contains contradictory statements regarding gold standard assessment:

"Individuals who presented a clinical speech-language diagnosis of dysphagia were referred for confirmation..."

"150 (32.9%) were confirmed to have dysphagia by videofluoroscopy"

Required clarifications:

How many of the 455 participants actually underwent videofluoroscopic swallowing study (VFSS)?

Was VFSS performed only on PARD-positive cases? If so, this constitutes verification bias, artificially inflating reported sensitivity and specificity.

Provide a CONSORT-style flow diagram detailing participant enrollment, screening outcomes, and gold standard assessment.

5. Machine Learning Methodology

Hyperparameter selection: Table 1 presents hyperparameters without justification (grid search, random search, or empirical selection?). The CNN architecture (single convolutional layer) is unusually simple for structured data; rationale for this choice versus traditional approaches is needed.

Data leakage risk: The description states: "The dataset was initially split... allocating 25% for testing and 75% for training," followed by "5-fold cross-validation (CV) on the training set." Critical ambiguity remains: Was SMOTE applied before CV or within each CV fold? Application before CV creates data leakage and overly optimistic performance estimates.

Correct pipeline: Raw data → Train/test split → CV on training data only → SMOTE applied to each training fold separately → Final evaluation on untouched test set.

6. Model Selection Criteria

The CNN was selected as the "best model" despite reduced sensitivity (0.84 vs. 0.87 for Naive Bayes and SVM). For public health screening applications, sensitivity prioritization is paramount—the cost of false negatives (missed dysphagia leading to aspiration pneumonia, malnutrition) substantially exceeds false positives. The model selection rationale should be reconsidered, or the advantages of Naive Bayes for sensitivity-prioritized screening should be discussed.

7. Interpretability Analysis

Current LIME explanations are limited to two true-positive cases. Missing are:

False positive and false negative case analyses (essential for understanding failure modes)

Global feature importance measures (permutation importance, SHAP values) rather than solely local explanations

8. Inadequate Limitations Discussion

Current limitations (sample size, convenience sampling) are insufficient. Required additions:

Verification bias risk (as detailed above)

Single-center limitation: All data from one Brazilian hospital; generalizability to diverse populations and healthcare systems is unknown

Temporal factors: Data collection spanned December 2021 to January 2025, encompassing COVID-19 pandemic periods that may affect variables such as COVID-19 history

Absence of external validation on independent datasets

Lack of prospective assessment in real-world screening workflows

9. Reporting Guidelines

Explicit statement of adherence to STARD 2015 (Diagnostic Accuracy Studies) is required—Reference 35 is cited but guideline compliance is not declared. For machine learning components, TRIPOD-ML or CLAIM guidelines should also be referenced.

Confidential Remarks to the Editor

The citation error in Reference 32 is highly concerning. The complete mismatch in authors, journal, year, and volume suggests either deliberate fabrication or unverified AI-generated content. This requires thorough investigation before any revision can be considered. Additionally, the potential verification bias in gold standard assessment represents a fundamental threat to the study's validity that must be addressed with transparent reporting of the actual VFSS completion rate.

**Reviewer #2:**  Methodology and Discussion: I suggest that the authors revise the manuscript to improve clarity and provide a more detailed description of the algorithms used. It is important to clearly explain how each model processes the data and what its main characteristics are, especially for those that achieved the best results (Naive Bayes, Support Vector Machine, and Convolutional Neural Network). In addition, considering that Convolutional Neural Networks are more commonly applied to complex data such as images and videos, it would be relevant to discuss why this model showed better performance in a structured tabular clinical dataset.

A clearer explanation of the relationship between the algorithms and the type of data used would help readers better understand the methodological choices and the results obtained.

**********

what does this mean?). If published, this will include your full peer review and any attached files.

**Do you want your identity to be public for this peer review?**  If you choose “no”, your identity will remain anonymous but your review may still be made public.

For information about this choice, including consent withdrawal, please see our Privacy Policy

Reviewer #1: No

Reviewer #2: **Yes:**  Rayane Délcia da Silva

**********

**Figure resubmission:**

**Reproducibility:**

To enhance the reproducibility of your results, we recommend that authors of applicable studies deposit laboratory protocols in protocols.io, where a protocol can be assigned its own identifier (DOI) such that it can be cited independently in the future. Additionally, PLOS ONE offers an option to publish peer-reviewed clinical study protocols. Read more information on sharing protocols at https://plos.org/protocols?utm_medium=editorial-email&utm_source=authorletters&utm_campaign=protocols

Attachment Submitted filename: PDIG-D-26-00067.docx

# Author comment: Author response to Decision Letter 1

**DOI:** 10.1371/journal.pdig.0001755.r002

15 May 2026

Attachment Submitted filename: Response to Reviewers.pdf

# Peer review: Decision Letter 1

**DOI:** 10.1371/journal.pdig.0001755.r003

**Authors:** Saskia Haitjema, Leo Anthony Celi

22 Jun 2026

Response to Reviewers Revised Manuscript with Track Changes Manuscript **Journal Requirements:**

**Additional Editor Comments (if provided):** **Reviewers' Comments:**

**Comments to the Author**

Reviewer #1: All comments have been addressed

Reviewer #2: All comments have been addressed

**********

publication criteria?

Reviewer #1: No

Reviewer #2: Yes

**********

3. Has the statistical analysis been performed appropriately and rigorously?

Reviewer #1: No

Reviewer #2: I don't know

**********

4. Have the authors made all data underlying the findings in their manuscript fully available (please refer to the Data Availability Statement at the start of the manuscript PDF file)?

The PLOS Data policy

Reviewer #1: No

Reviewer #2: Yes

**********

5. Is the manuscript presented in an intelligible fashion and written in standard English?

Reviewer #1: Yes

Reviewer #2: Yes

**********

**Reviewer #1:**  3. Sampling, Sample Size, and Missing Data

Authors' response: The authors expanded the Discussion on convenience sampling limitations, acknowledged the lack of a priori sample size calculation, and explicitly reported class distributions before/after SMOTE and across train/test splits.

Referee comment: The reporting of class distribution and SMOTE application is now transparent. However, missing data handling still lacks rigor: the 43 excluded observations were removed without reporting missingness patterns (e.g., MCAR vs. MAR) or conducting sensitivity analyses to validate the deletion approach. Given that 36 of the 42 exclusions were due to missing O'Neill Scale data (per Figure 2), this is not a trivial issue. The authors are advised to add a brief missingness analysis.

4. Critical Methodological Flaw: Verification Bias ❌

Authors' response: The authors provided a STARD-based participant flow diagram (Figure 2) and stated: "Videofluoroscopy was used as the reference standard for diagnosing dysphagia... All assessments were performed with blinding between the index test and the reference standard."

Referee comment: This is the most serious unresolved issue in the revised manuscript, and paradoxically, the newly added flow diagram exposes the problem more clearly than the original text:

• Of 497 eligible participants, only 288 underwent VFSS (the reference standard).

• 209 participants (22 with suspected dysphagia but PARD-only, and 187 with no suspected dysphagia and PARD-only) did not undergo VFSS.

• Yet the final analytic dataset of 455 participants is dichotomized into 150 "Dysphagia" and 305 "No Dysphagia."

The authors never clarify how the 305 "non-dysphagia" cases were verified. If a substantial proportion were classified as negative based solely on PARD (a screening tool, not a gold standard), this constitutes severe partial verification bias. In diagnostic accuracy studies, using the screening test's negative result to define the reference-standard negative class artificially inflates both specificity and accuracy, while the true sensitivity remains unknown. This issue is unacceptable and must be fully resolved.

5. Machine Learning Methodology ⚠️

Authors' response: The authors stated that hyperparameters were selected based on literature defaults and exploratory experiments (without formal grid/random search), justified the simple CNN architecture for tabular data, and claimed to have fixed the SMOTE-CV pipeline so that SMOTE is applied exclusively within each training fold.

Referee comment: While the methodological descriptions are now clearer, the revised results differ so dramatically from the original manuscript that they raise serious concerns about the integrity of the original analysis:

| Model | Original Test Sensitivity | Revised Test Sensitivity |

|:---|:---|:---|

| Naive Bayes | 0.87 | 0.74 |

| SVM | 0.87 | 0.76 |

| CNN | 0.84 | 0.76 |

| Random Forest | 0.58 | 0.71 |

Such large shifts, particularly the collapse of NB and SVM performance, and the simultaneous improvement of RF, cannot be explained solely by correcting the SMOTE-CV sequence. They strongly suggest that the original manuscript contained additional data leakage, coding errors, or analytical mistakes that have now been (partially) corrected. Furthermore, the hyperparameters themselves have changed (e.g., LR: C from 1 to 0.01, max_iter from 200 to 1000; MLP: max_iter from 300 to 1000). The authors do not explain why these changes were necessary alongside the pipeline fix.

Serious doubt must be raised. The authors should provide a detailed explanation and revision, and must release the complete analysis code (Python scripts) to the journal for independent verification.

6. Model Selection Criteria

Authors' response: The authors revised the Discussion to acknowledge that sensitivity should be prioritized in screening contexts. They now highlight the KNN model (sensitivity 0.79) as a viable option despite its lower overall metrics, and discuss the trade-off between sensitivity and specificity.

Referee comment: The response is conceptually appropriate. However, the revised maximum sensitivity of 0.79 (KNN) is notably lower than the originally claimed 0.87. The authors should more cautiously qualify the statement that the results demonstrate "adequate diagnostic accuracy," as a sensitivity of 0.79 for a screening tool, while better than RF's 0.71, still implies that 21% of true dysphagia cases would be missed. In a public health screening context, the wording should be more cautious and restrained.

8. Inadequate Limitations Discussion

Authors' response: Added discussion of single-center design, COVID-19 temporal factors, lack of external validation, and need for prospective assessment.

Referee comment: These additions are welcome. However, the most fundamental limitation: verification bias (Point 4 above), remains entirely unmentioned and should be emphasized. The authors cannot claim methodological transparency while omitting discussion of the fact that a large proportion of "non-dysphagia" cases were never verified by the reference standard. This omission must be corrected.

New Concerns Arising from the Revision

1. Performance inconsistency: The dramatic decline in model performance after pipeline correction (Point 5) undermines confidence in the original findings and requires code-level verification.

2. Flow diagram logic: The STARD flow diagram (Figure 2) inappropriately includes participants who never underwent the reference standard (VFSS) in a diagnostic accuracy "Complete Dataset." This is methodologically incorrect and must be either redrawn with separate pathways for VFSS-verified vs. non-verified participants, or accompanied by explicit statistical handling of the verification bias.

Final Recommendation

Major Revision

While the authors have made genuine efforts to improve transparency and reporting, the revised manuscript fails to resolve its most critical methodological flaw: the verification bias arising from the fact that a substantial proportion of participants classified as "non-dysphagia" never underwent the reference standard (VFSS). The STARD flow diagram, intended to clarify the study workflow, instead makes this problem visually obvious. Additionally, the drastic change in model performance after analytical corrections raises serious questions about the reliability of the original results that the authors have not adequately explained.

To proceed, the authors must:

1. Clarify exactly how many of the 455 analyzed participants underwent VFSS, and how the remaining participants were classified.

2. If partial verification exists, provide a sensitivity analysis using only VFSS-completed participants and add an explicit discussion of verification bias to the Limitations.

3. Explain the dramatic performance shifts and provide analysis code for independent verification.

Without these clarifications, the diagnostic accuracy claims in this manuscript remain unsubstantiated.

**Reviewer #2:**  (No Response)

**********

what does this mean?). If published, this will include your full peer review and any attached files.

**Do you want your identity to be public for this peer review?**  If you choose “no”, your identity will remain anonymous but your review may still be made public.

For information about this choice, including consent withdrawal, please see our Privacy Policy

Reviewer #1: **Yes:**  Huan Du

Reviewer #2: No

**********

**Figure resubmission:**

**Reproducibility:**

To enhance the reproducibility of your results, we recommend that authors of applicable studies deposit laboratory protocols in protocols.io, where a protocol can be assigned its own identifier (DOI) such that it can be cited independently in the future. Additionally, PLOS ONE offers an option to publish peer-reviewed clinical study protocols. Read more information on sharing protocols at https://plos.org/protocols?utm_medium=editorial-email&utm_source=authorletters&utm_campaign=protocols

# Author comment: Author response to Decision Letter 2

**DOI:** 10.1371/journal.pdig.0001755.r004

21 Jul 2026

Attachment Submitted filename: Response_to_Reviewers.pdf

# Peer review: Decision Letter 2

**DOI:** 10.1371/journal.pdig.0001755.r005

**Authors:** Saskia Haitjema, Leo Anthony Celi

8 Sep 2026

Artificial Intelligence for Dysphagia Screening: A Machine Learning Approach

PDIG-D-26-00067R2

Dear Dr. Hugo,

We are pleased to inform you that your manuscript 'Artificial Intelligence for Dysphagia Screening: A Machine Learning Approach' has been provisionally accepted for publication in PLOS Digital Health.

Before your manuscript can be formally accepted you will need to complete some formatting changes, which you will receive in a follow-up email from a member of our team.

Please note that your manuscript will not be scheduled for publication until you have made the required changes, so a swift response is appreciated.

IMPORTANT: The editorial review process is now complete. PLOS will only permit corrections to spelling, formatting or significant scientific errors from this point onwards. Requests for major changes, or any which affect the scientific understanding of your work, will cause delays to the publication date of your manuscript.

If your institution or institutions have a press office, please notify them about your upcoming paper to help maximize its impact. If they'll be preparing press materials, please inform our press team as soon as possible -- no later than 48 hours after receiving the formal acceptance. Your manuscript will remain under strict press embargo until 2 pm Eastern Time on the date of publication. For more information, please contact digitalhealth@plos.org.

Thank you again for supporting Open Access publishing; we are looking forward to publishing your work in PLOS Digital Health.

Best regards,

Saskia Haitjema, MD

Academic Editor

PLOS Digital Health

***********************************************************

**Additional Editor Comments (if provided):**

**Reviewer Comments (if any, and for reference):**

Reviewer's Responses to Questions

**Comments to the Author**

Reviewer #1: All comments have been addressed

**********

publication criteria?

Reviewer #1: Yes

**********

3. Has the statistical analysis been performed appropriately and rigorously?

Reviewer #1: Yes

**********

4. Have the authors made all data underlying the findings in their manuscript fully available (please refer to the Data Availability Statement at the start of the manuscript PDF file)?

The PLOS Data policy

Reviewer #1: Yes

**********

5. Is the manuscript presented in an intelligible fashion and written in standard English?

Reviewer #1: Yes

**********

Reviewer #1: Reviewer’s Decision Letter

To the Editor, PLOS Digital Health

Manuscript ID: PDIG-D-26-00067R2

Title: Artificial Intelligence for Dysphagia Screening: A Machine Learning Approach

Decision: Minor Revision

Overall Assessment

The authors have made a substantial and commendable effort to address the concerns raised in the previous review round. The R2 revision demonstrates genuine engagement with the most critical issues, particularly the verification bias concern and the unexplained performance shifts between analyses.

The revised manuscript now includes: (1) a transparent description of the two-stage outcome ascertainment procedure, with explicit distinction between VFSS-verified and clinically-classified participants; (2) a comprehensive sensitivity analysis restricted to the VFSS-confirmed cohort (n=290), with results reported across multiple algorithms and both with and without SMOTE; (3) an expanded Limitations section that explicitly acknowledges verification bias as a fundamental limitation; and (4) an offer to release the complete analysis code for independent verification.

The authors' response to the performance shift concern is credible: the R2 analysis represents a complete rerun of the computational workflow, involving not only the SMOTE-CV correction but also a systematic review and standardization of model-specific configurations. The provision of analysis code for audit is the appropriate remedy.

One issue remains that requires attention before acceptance: the verification bias, while now transparently disclosed and subjected to sensitivity analysis, still affects the interpretability of the primary performance estimates. The authors should provide a clearer, quantified discussion of how the sensitivity analysis results differ from the primary analysis, and what this implies for the clinical interpretation of the model performance. This can be addressed through textual revision without additional experiments.

I therefore recommend minor revision.

Comments for the Authors

1. Verification bias – provide a more quantified comparison between primary and sensitivity analyses

The authors have done an excellent job in acknowledging and analyzing verification bias. The sensitivity analysis restricted to the 290 VFSS-confirmed participants is a rigorous addition. However, the Discussion currently presents the sensitivity analysis results in qualitative terms ("overall performance... was lower than that observed in the primary analysis, consistent with the substantially smaller sample size"). This does not fully convey the clinical implications of the bias.

Recommendation: Add a brief, quantified statement comparing the key performance metrics (e.g., sensitivity, specificity, accuracy, MCC) between the primary analysis (n=477) and the VFSS-only analysis (n=290) for the best-performing model(s) – preferably Logistic Regression or Naive Bayes. This will help readers understand the magnitude of potential overestimation due to verification bias. For example: "For Logistic Regression, sensitivity decreased from 0.82 in the primary analysis to 0.63 in the VFSS-confirmed cohort, while specificity decreased from 0.77 to 0.69." Such a comparison would provide a more transparent assessment of the bias's impact.

2. Performance shift – confirm code availability for review

The authors have stated that "the complete analysis code has been included with the revised submission for the reviewers' audit." This is the appropriate response to the concern about performance inconsistency. Please confirm that the code is indeed accessible and that it reproduces the reported results. If not already done, the authors should ensure the code is well-documented enough for an independent reviewer to verify the analytical pipeline.

Recommendation: The authors should confirm in their response that the code is fully reproducible and, if possible, provide a brief README or instructions for running the analysis. This is not a requirement for publication but would significantly strengthen transparency.

3. Missing data – minor clarification on exclusion rationale

The authors have explained that missing O'Neill Scale data were procedural (VFSS reports not yet issued at the time of dataset compilation) and most consistent with MAR. This is a reasonable justification. However, the manuscript states that "the best approach is to exclude these observations" without elaborating on why multiple imputation or other missing data methods were not considered.

Recommendation: Add a brief sentence explaining why listwise deletion was preferred (e.g., small proportion of missing data, or the MAR assumption being reasonable given the procedural nature of missingness). This is a minor clarification.

4. Wording of conclusions – maintain the cautious tone

The authors have appropriately toned down the claims in the Discussion and Conclusion. I appreciate the revised language: "These models should not replace clinical or instrumental swallowing assessments." and "the findings should be considered exploratory." Please ensure this cautious tone is maintained throughout, particularly in the Abstract where the phrase "adequate diagnostic accuracy" still appears.

Recommendation: In the Abstract, consider revising "demonstrating adequate diagnostic accuracy" to "demonstrating exploratory diagnostic accuracy that warrants further validation" or similar phrasing to maintain consistency with the Discussion's cautious framing.

Conclusion

The authors have responded thoroughly to the previous review concerns. The verification bias is now transparently disclosed and analyzed; the performance shift is explained and accompanied by code availability; the limitations discussion is substantially expanded; and the framing is appropriately cautious. The remaining issues are minor and can be addressed through textual clarification without additional experiments. I recommend acceptance after minor revisions.

Sincerely,

Reviewer

**********

what does this mean?). If published, this will include your full peer review and any attached files.

**Do you want your identity to be public for this peer review?**  If you choose “no”, your identity will remain anonymous but your review may still be made public.

For information about this choice, including consent withdrawal, please see our Privacy Policy

Reviewer #1: **Yes:**  Huan Du

**********

Attachment Submitted filename: renamed_a6801.docx
